# Workflow Topology–Guided Skill Loading for Agentic AI in Clinical Trial Data Pipelines: Sequential Exploratory Evaluation

**DOI:** 10.2196/100769

**Published:** 2026-10-20

**Authors:** Jaime Yan

**Affiliations:** 1 Department of Data Science Harrisburg University of Science and Technology Harrisburg, PA United States

**Keywords:** AI, large language model, clinical trials, CDISC, clinical data interchange standards consortium, context engineering, knowledge graph, agentic AI, retrieval, domain adaptation

## Abstract

**Background:**

Clinical trial data pipelines must follow Clinical Data Interchange Standards Consortium (CDISC) standards, including the study data tabulation model (SDTM) and analysis data model (ADaM). General-purpose large language models (LLMs) can produce plausible artifacts that omit required variables, misuse terminology, or invent derivation logic. Curated context may reduce these errors, but the value of organizing that context by workflow topology has not been separated from corpus access alone.

**Objective:**

This study aimed to examine when workflow topology–guided skill loading adds value beyond a domain prompt, nonrouted curated context, or lexical retrieval, and whether bounded scheduling retains quality with less injected context.

**Methods:**

A 45-node, 7-layer directed acyclic workflow graph was linked to 59 modular rule files (SKILL.md). A sequential exploratory evaluation examined 4 evidence regimes spanning domain-context, component isolation, lexically reachable, and topology-dependent conditions (10 tasks, 90 runs; 10 tasks, 180 and 120 runs; 24 tasks, 288 runs; and 18 tasks, 324 runs; the task, not the agent run, was the inferential unit). End points included LLM rubric scores, deterministic atomic criteria, required-evidence recall, and manifest checks. A blinded practicing statistical programmer independently rescored 60 outputs. Task-level paired inference used bootstrap 95% CIs, exact sign-flip tests, and Holm adjustment.

**Results:**

Routed context scored 4.63 versus 4.20 for prompt-only (difference 0.43 on a 1-5 scale; 95% CI 0.25-0.63; Holm-adjusted *P*=.01) but did not exceed flat or random loading. The lower-context scheduler used 28.82% less context while retaining quality; all 559 injected fragments had recorded sources, and all 120 manifests were reproduced exactly. On lexically reachable tasks, graph+Best Match 25 (BM25) and BM25-only were equivalent within a 0.05 margin (difference +0.009; 95% CI –0.028 to 0.046; *P*=.67), where BM25 already recalled 95.8% of required evidence. On topology-dependent stress tasks, graph-only selection outperformed BM25-only (difference 0.348; 95% CI 0.178-0.530; nominal *P*=.002; *d*_z_=0.89) and raised required-evidence recall from 0.500 to 1.000. Expert-judge agreement was substantial for terminology (weighted κ=0.61) and moderate overall (intraclass correlation coefficient=0.60).

**Conclusions:**

Workflow topology serves as a conditional retrieval prior. It adds little when lexical retrieval already reaches the required evidence but recovers dependency-linked evidence when task wording does not identify it lexically. These findings, from a synthetic benchmark using one generator backbone and one expert rater, support topology-guided selection as a promising approach for controlled context assembly and motivate independent factorial validation.

## Introduction

### Background

AI has advanced clinical research and care delivery in several domains [1-4], yet its application to the regulatory data pipeline remains underexplored. Clinical trial data submitted to regulators must be represented using defined study data standards and implementation guides [5-9]. Preparing study data tabulation model (SDTM) domains, analysis data model (ADaM) datasets, tables, figures, listings, and Define-XML metadata requires specialized data management and statistical programming. A generated artifact can look plausible while still containing incorrect controlled terminology, missing population flags, invalid derivations, or incomplete traceability.

Agentic AI systems can combine language model generation with tools and external knowledge [10,11], but unconstrained systems can hallucinate terminology and procedural details [12-15]. Systematic benchmarking has begun to characterize both the capabilities and the performance variability of clinical AI systems [16-18], and machine learning has shown broad applicability across the drug development lifecycle [19]. General fine-tuning and broad prompting may not be sufficient for work that depends on stage-specific rules and ordered validation steps [20]. Retrieval-augmented generation (RAG) can ground outputs in external documents [21,22], yet ordinary lexical retrieval does not explicitly represent which upstream or downstream workflow stages contain evidence needed by the current task.

### Workflow Topology as a Retrieval Prior

The mechanism studied here is narrower than a general agent framework or knowledge base. The clinical evidence pipeline is represented as a directed workflow graph. Rule files are bound to graph nodes, and the active node and its dependencies determine which files are candidates for the model’s limited context. Workflow topology can therefore contribute information that is absent from the task wording. The two rule files may be related because one stage requires, enables, or validates another, even when their headings share few terms.

This mechanism should not be expected to help equally in every task. If a prompt names the exact node and required section, full-corpus lexical retrieval may already recover the evidence. In that regime, graph traversal can improve control or traceability without changing output quality. If the prompt omits the relevant terminology but the workflow dependency identifies the needed evidence, topology may recover material that lexical retrieval misses. This conditional hypothesis—not universal graph superiority—organizes the present evaluation.

### Relationship to Prior Work

General agent frameworks provide orchestration primitives [23-25], while knowledge graph and RAG research addresses external grounding and structured relations [21,22,26]. The foundational Constrained Skill Priority (CSP)–Engine architecture, including its workflow graph and scheduling concept, was previously released as open-source software with accompanying documentation [27]. The present article contributes the empirical evaluation. Specifically, it adds (1) controlled comparisons separating a domain prompt, access to a curated rule corpus, and topology-guided selection; (2) analysis of 2 deliberately different evidence regimes—one lexically reachable and one dependency-linked but lexically mismatched; (3) measurements of context volume and manifest-level provenance, reproducibility, and change scope; and (4) an evaluation audit combining complete-response LLM scoring, deterministic criteria, presentation-order diagnostics, and blinded concordance with a practicing statistical programmer. The experimental results reported here are new to this article.

### Objective

We asked, “Under what task and evidence conditions does workflow topology add value beyond a domain prompt, nonrouted curated context, or lexical retrieval?” A secondary objective was to determine whether bounded scheduling could retain benchmark quality with less injected rule context and stronger manifest-level controls. The study was exploratory and sequential; each phase was designed to probe the boundary conditions surfaced by the preceding phase.

## Methods

### Study Design

We conducted a sequential exploratory evaluation of AI-generated regulatory programming artifacts on synthetic tasks. The sequence was adaptive. The later phases were not all prospectively locked at the outset. We report the work as a single evidence chain in which each phase isolates a distinct condition under which topology might—or might not—add value.

The 4 phases had distinct scientific roles (Table 1). The domain-context phase tested a bundled domain prompt plus node-bound curated rules against a bare agent. The component and efficiency phase separated prompt, corpus access, routing, and distilled principles and then tested whether lower-context scheduling retained quality while recording manifest-level governance properties. The lexically reachable phase used deterministic criteria to compare dependency-aware graph-plus Best Match 25 (BM25) retrieval with full-corpus BM25 on tasks whose required evidence was usually named in the prompt. The topology-dependent stress phase tested pure graph selection on constructed tasks whose required evidence lay in a related workflow node and was deliberately lexically mismatched with the prompt.

An adapted Transparent Reporting of a Multivariable Prediction Model for Individual Prognosis or Diagnosis—AI (TRIPOD-AI) checklist is provided as a transparency aid in Multimedia Appendix 1; it is not a claim of prediction-model reporting compliance. Multimedia Appendix 2 retains the chronological implementation identifiers and experiment-refinement log for reproducibility without using those identifiers as the scientific narrative.

**Table 1:** Sequential evidence phases and their identifying roles.

Phase	Task panel and runs	Main comparison	What the phase can identify
Domain-context	10 tasks, 3 arms, 3 replicates; 90 runs	Domain prompt+node-bound rules vs bare agent	Effect of the bundled intervention; not a graph effect
Component and efficiency	10 tasks; 180-run, 6-arm comparison and 120-run, 4-arm comparison	Routed vs prompt-only, flat, and random context	Corpus/routing separation on the standard panel; context-retention and manifest properties
Lexically reachable evidence	24 held-out tasks, 4 arms, 3 replicates; 288 runs	Graph+BM25^a^ vs token-matched full-corpus BM25	Incremental topology value when lexical retrieval is already strong
Topology-dependent evidence	18 constructed tasks, 6 arms, 3 replicates; 324 runs	Graph-only vs BM25-only	Mechanism behavior when graph dependencies, rather than prompt words, identify evidence

^a^BM25: Best Match 25.

### Regulatory Workflow Graph

The clinical trial data pipeline was represented as a directed acyclic graph, *G*=(V,E), comprising 45 nodes across 7 procedural layers (Figure 1). Nodes represented tasks such as adverse-event mapping, SDTM validation, ADaM derivation, and Define-XML assembly. Three edge types encoded required ordering, validation gates, and data flow. The complete node inventory and edge relationships are provided in Multimedia Appendix 3. The graph is a simplified model; real submissions include iterative validation and metadata loops that a directed acyclic representation linearizes.

**Figure 1:**
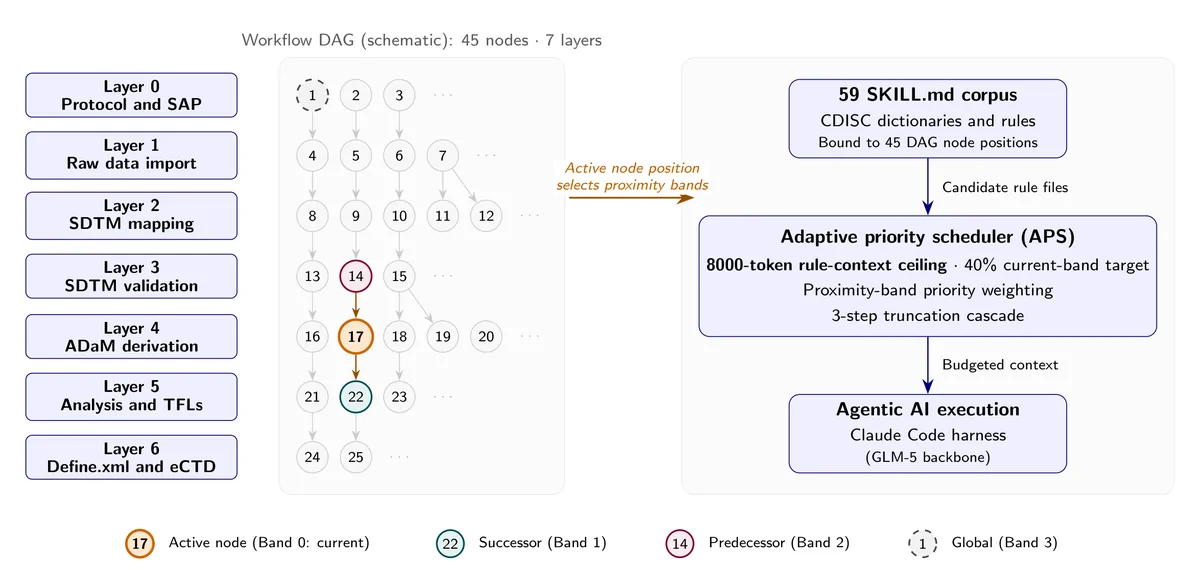
Constrained Skill Priority (CSP)–Engine architecture (schematic; a subset of the 45 nodes is shown). A 45-node clinical trial data workflow spans 7 layers. The active node and adjacent predecessor, successor, and global nodes define candidate SKILL.md rule files. The Adaptive Priority Scheduler ranks and truncates candidates within an 8000-token rule-context ceiling toward a 40% current-band target before passing the selected context and task to the Claude Code harness with a General Language Model 5 (GLM-5) backbone. ADaM: analysis data model; APS: Adaptive Priority Scheduler; CDISC: Clinical Data Interchange Standards Consortium; DAG: directed acyclic graph; eCTD: electronic Common Technical Document; GLM-5: General Language Model 5; SAP: statistical analysis plan; SDTM: study data tabulation model; TFL: tables, figures, and listings.

### Rule Corpus and Distilled Principles

SKILL.md is a convention for a modular Markdown-based rule file that binds a bounded unit of domain knowledge to a workflow position. We authored 59 files containing Clinical Data Interchange Standards Consortium (CDISC) terminology, procedural derivations, input/output specifications, pseudocode, and validation criteria (Multimedia Appendix 4). Normative sources were SDTMIG version 3.4 [5], ADaMIG version 1.3 [6], and Define-XML version 2.1 [7] and associated controlled terminology. Each rule was cross-checked against the corresponding source section and exercised against synthetic fixtures. The corpus was authored and checked by one author and was not independently audited by external CDISC experts.

An exploratory “distilled principles” store contained 50 short principles generated by a large language model (LLM) from successful and unsuccessful pilot trajectories. The 2 principles ranked most relevant to a task were appended to its context. No redundancy or conflict-pruning step compared these principles with local rule files.

### Adaptive Priority Scheduler

The Adaptive Priority Scheduler (APS) operated under an 8000-token rule-context ceiling. Candidate files were assigned to current, successor, predecessor, or global bands with weights 1.0, 0.6, 0.4, and 0.2. Priority equaled band weight multiplied by 1 plus the hub bonus (0.03 per incident edge, capped at 0.3). Query similarity was not used in the scheduler. Ties were resolved alphabetically. After reservations, the scheduler served current-band demand toward a 40% target and then processed remaining candidates greedily. Each non-current-band file was capped at 25% of the available budget. Oversized files passed through a deterministic cascade that removed front matter, removed example code blocks, and then applied a character cutoff. Edge type determined adjacency but did not add a separate weight. Multimedia Appendix 5 provides implementation-matched pseudocode.

The domain-context phase used node-bound rule injection without banded scheduling; the full APS was introduced in the component comparison phase. This design choice means that the first phase provides bundled intervention evidence, not topology-weighted scheduling evidence.

The later efficiency configuration preserved all current-band content, admitted non-current-band content using deterministic task relevance, and imposed a 6720-token soft cap (84% of the common ceiling), with an override for complete current-band demand up to 8000 tokens. The lower-context volume was thus partly policy-enforced; preservation of benchmark quality was the empirical question.

### Experimental Conditions

All generation conditions used the same Claude Code (Anthropic) command-line harness, which routed requests to a GLM-5 backbone. Runs used isolated working directories, frozen task prompts, and recorded configurations. Computational environment details and recorded token-cost summaries are provided in Multimedia Appendix 6. The number of agent runs describes the amount of computation; replicates did not increase the inferential task count.

### Domain-Context Phase

Ten tasks spanned 6 regulatory layers. Three conditions were run 3 times per task: a bare agent with no domain system prompt or rule files; a domain prompt with node-bound rules condition; and the same condition plus 2 distilled principles. This phase used 90 runs. Because prompt and rule access changed together, this phase cannot isolate the effect of the graph.

### Component and Efficiency Phase

The 6 conditions were bare agent, domain prompt only, APS, APS plus 2 principles, budget-capped flat loading with uniform alphabetical priority, and seeded random loading of 5 files. Three replicates of each task-condition cell produced 180 runs. The random arm matched file count, not context tokens.

Following the component comparison, a 4-arm efficiency comparison used frozen APS, the lower-context scheduler, flat loading at 8000 tokens, and seeded random loading matched to the lower-context arm’s realized volume. The same 10 tasks and 3 replicates produced 120 runs. Prespecified engineering retention required the lower bound of the 95% CI for the lower-context-versus-flat comparison to be at least –0.20 on the 1-5 scale, at least 15% fewer rule-context tokens, complete fragment-source linkage and manifest reproduction, and no broader mean simulated change impact than flat loading. The –0.20 threshold was an engineering decision rule, not a validated clinical noninferiority margin.

### Lexically Reachable Evidence Phase

Twenty-four new tasks were scored with machine-verifiable atomic criteria covering required variables, controlled terminology versions, forbidden terms, derivation formulas, schema validity, evidence identifiers, and upstream change propagation. The primary end point was the fraction of weighted criteria passed (0-1); an LLM readability score was secondary.

Four section-chunk retrieval arms were evaluated: dependency-aware graph expansion followed by BM25 scoring; full-corpus BM25 with no graph; seeded random retrieval; and flat loading at 8000 tokens. Graph+BM25, BM25-only, and random retrieval used exactly matched task-specific canonical-token budgets. Three replicates produced 288 runs. The primary comparison family comprised graph+BM25 versus BM25-only and graph+BM25 versus random, with Holm adjustment. A supporting flat comparison was governed by the retention rule. The router and task definitions were frozen internally before generation; the study was not publicly preregistered.

An offline evidence audit subsequently measured whether registry-required sections were present in each context and classified failures as retrieval-side or generation-side. This diagnostic was used to interpret benchmark sensitivity, not to change the primary end point.

### Topology-Dependent Evidence Stress Phase

The final phase used 18 author-constructed tasks in which required evidence was located at least 1 workflow hop from the active node, spanned dependency-linked nodes, and had deliberately low lexical overlap with the prompt. Six arms were executed: graph-only; graph-only with typed-edge priority; graph expansion followed by BM25; full-corpus BM25; flat loading at 8000 tokens; and seeded random loading. Three replicates produced 324 runs.

Graph-only ranked reachable skills by hop band and deterministic round-robin/document order without a BM25 term. The deterministic 0-1 score combined one substantive output criterion with a required-evidence/provenance criterion. No LLM judge was used. Required-evidence recall—the proportion of tasks whose prespecified source section appeared in the injected context—was reported as a separate mechanism outcome.

This phase was designed as a routing-hostile stress test after an offline oracle had established arm separation. It was not a clean factorial intervention: graph-only and BM25-only used different admission policies and mean context volumes (2157 vs 4000 canonical tokens); arms ran in a fixed order; 6 task prompts shared 2 evidence targets, leaving 14 unique targets; and the author aligned the graph, tasks, source sections, and criteria. Raw response-level criterion records were not retained in the result bundle. Consequently, graph-only versus BM25-only is a descriptive policy contrast under constructed fixed conditions, not an isolated causal graph main effect or confirmatory population estimate.

### Evaluation Instruments

The evaluation hierarchy is summarized in Table 2. LLM scores, deterministic scores, retrieval recall, and manifest checks measure different constructs; their numerical effects are not interchangeable.

**Table 2:** Evaluation instruments and claim boundaries.

Instrument	Scale/unit	Role	Principal limitation
Complete-response LLM^a^ rubric	Structure, terminology, completeness; 1-5	Primary outcome in the first 2 phases	Model/version dependence
Persona-conditioned LLM panels	1-5 plus pairwise choice	Reliability and qualitative interpretation	Automated raters are not domain experts
Deterministic atomic criteria	Weighted fraction passed; 0-1	Primary outcome in retrieval phases	Criteria and weights were author-designed
Required-evidence recall	Source section present/absent	Mechanism diagnostic	Presence does not guarantee correct use
Manifest checks	Counts, hashes, simulated scope	Architecture/governance proxies	Not operational good practice (GxP) validation
Expert concordance	Kappa, ICC^b^, contrast direction	Limited judge-calibration evidence	One rater and one seeded output per cell

^a^LLM: large language model.

^b^ICC: intraclass correlation coefficient.

In the domain-context and component phases, complete stored responses were scored with a pinned, temperature-0 DeepSeek-V4-Flash (DeepSeek; API model identifier deepseek-v4-flash) JSON protocol, following the LLM-as-a-judge methodology introduced by Zheng et al [28]; scoring covered each full response rather than a truncated prefix, because response length varied widely and a 4000-character cap would have truncated 57/90 domain-context and 158/180 component responses. The subsequent context-efficiency and lexically reachable phases used a pinned DeepSeek-V4-Pro judge (API model identifier deepseek-v4-pro) for scalar and readability scoring; recorded model identifiers, system fingerprints, and per-call timestamps for both judge models are preserved in the versioned archival snapshot described in the Data Availability section. Bidirectional pairwise judging was retained only as an order-sensitivity diagnostic because 9/30 domain-context pairs and 58/90 component pairs were order-discordant. Full scoring prompts, persona instructions, and randomization materials are provided in Multimedia Appendix 7; complete-response scoring outputs, task-paired analyses, order-sensitivity diagnostics, and provenance hashes are provided in Multimedia Appendix 8.

Two independent persona-conditioned LLM panels evaluated the bundled domain-context comparison from clinical data management, statistical programming, and regulatory perspectives; these were model simulations, not human experts. Separately, one practicing statistical programmer with 10 years of experience, blinded to condition, independently rescored one seeded stored output from each of the 60 component task-condition cells using the same 3D rubric. The condition key remained sealed until the scoring sheet was returned. The reported scoring time and absence of AI assistance were confirmed after scoring (Multimedia Appendix 9). This single-rater study assessed concordance with the automated judge; it did not validate regulatory compliance or establish professional consensus.

### Statistical Analysis

Replicates were averaged within each task-condition cell before inference, making task the unit of analysis. We report raw paired differences on the native outcome scale, paired-task bootstrap 95% CIs, exact 2-sided sign-flip *P* values, and paired standardized mean differences (*d*_z_) where available. The first 2 phases used 10,000 paired bootstrap resamples. The lexically reachable phase used 20,000 resamples. The topology-dependent stress-phase analysis used 10,000 resamples with seed 42 plus contrast index.

Holm adjustment was applied to the 3 prespecified component routing contrasts, the 3 efficiency contrasts, and the 2 lexically reachable routing contrasts. The topology-dependent stress phase had no prospectively locked multiplicity family. Its graph-only-versus-BM25 *P* value is therefore reported as nominal; a post hoc Holm adjustment across the 5 code-declared contrasts yielded *P*=.006 and is provided only as a sensitivity check.

Two one-sided tests (TOSTs) [29,30] were used post hoc to distinguish an undetected difference from practical similarity within explicit margins. Equivalence was concluded only when both one-sided tests were rejected at α=.05. These margins were chosen after the experiments and are interpretive diagnostics, not prospective noninferiority thresholds. Failure to establish equivalence was not treated as evidence that a difference exceeded the margin. Achieved-power calculations were used only to characterize design sensitivity.

### Ethical Considerations

The evaluation used no patients, human tissue, patient records, or identifiable personal information; all task fixtures were synthetic. Patient informed consent and privacy protections for participant data were not applicable. One practicing programmer acted as a professional annotator of model outputs. The study analyzed the annotations, not data about the annotator, and reports no personal information beyond professional experience. The author determined that this activity did not meet the US federal definition of human-subject research under 45 Code of Federal Regulations (CFR) 46.102 [31]; accordingly, no institutional review board application was submitted. This determination was reached by the author alone; the institution was not consulted. The broader governance framework for AI in health applications was considered [32]. Before agreeing to take part, the annotator was informed that the task involved independently scoring 60 blinded outputs of an AI system for clinical trial programming on a 3D anchored rubric; that no information about the generating system configurations would be provided and that the annotator should not guess them; that the ratings would be used solely to compare professional and automated judgments of output quality and would be reported only in aggregate; and that participation was voluntary. The annotator confirmed having no authorship or financial interest in the study before scoring and was not financially compensated for this work. Ratings are reported only in aggregate.

## Results

### Domain-Context Improved the Synthetic Benchmark, but the Bundled Comparison Did Not Identify Topology

In the complete-response reanalysis, the domain prompt with node-bound rules condition scored 4.47 versus 3.59 for the bare agent (difference of 0.88 on the 1-5 scale; 95% CI 0.63-1.13; exact *P*=.002; dz=2.07; Figure 2). Structure improved by 1.03, terminology by 0.83, and completeness by 0.77 (Holm-adjusted *P*=.006 for each). Per-task results are provided in Multimedia Appendix 10. The 2 independent persona panels produced a directionally consistent margin of 0.75 and showed substantial agreement with one another on pairwise choices (Cohen κ=0.76); persona-panel scores and interpanel agreement are provided in Multimedia Appendix 11.

**Figure 2:**
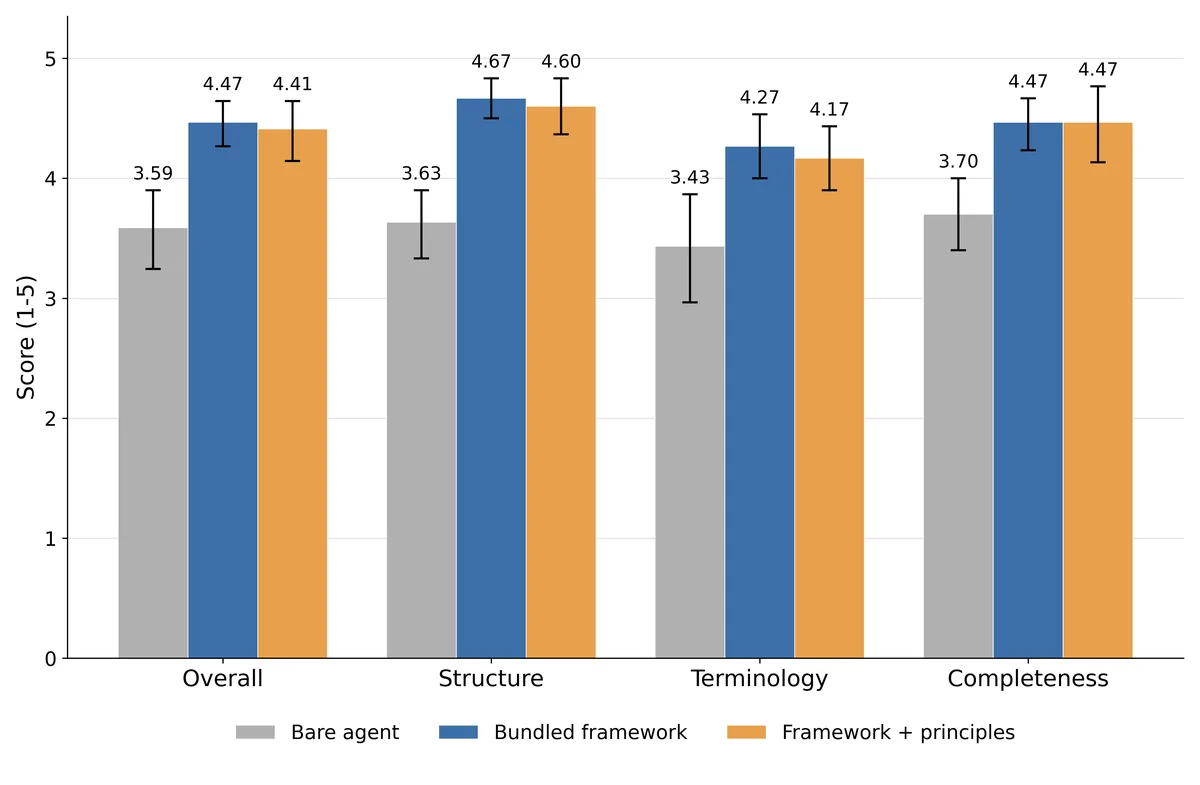
Complete-response domain-context phase. Mean task-level structure, terminology, completeness, and overall scores are shown on the 1-5 automated rubric for the bare agent and domain-prompt-plus-node-bound-rule conditions. Error bars are paired-task bootstrap 95% CIs.

In Figure 2, the comparison evaluates a bundled intervention and does not isolate workflow topology.

The condition combined a domain system prompt with access to node-bound rules, and the banded scheduler was not applied in this phase. The result therefore establishes only a bundled domain-context signal on this benchmark. It does not attribute the gain to workflow topology.

### Component Isolation and Context Efficiency

In the 6-condition comparison (Figure 3A; Multimedia Appendix 12), mean scores were 3.98 (SD 0.57) for the bare agent, 4.20 (SD 0.46) for domain prompt only, 4.44 (SD 0.45) for random loading, 4.58 (SD 0.29) for flat loading, 4.63 (SD 0.34) for APS, and 4.61 (SD 0.30) for APS plus principles. APS exceeded the domain-prompt-only condition by 0.43 (95% CI 0.25-0.63; exact *P*=.004; Holm-adjusted *P*=.01). It did not outperform flat loading (difference 0.05; 95% CI –0.18 to 0.33; exact and Holm-adjusted *P*=.78) or random loading (difference 0.19; 95% CI –0.05 to 0.46; exact *P*=.21; Holm-adjusted *P*=.42). Adding distilled principles changed the mean by –0.02 (95% CI –0.11 to 0.08; exploratory *P*=.83).

**Figure 3:**
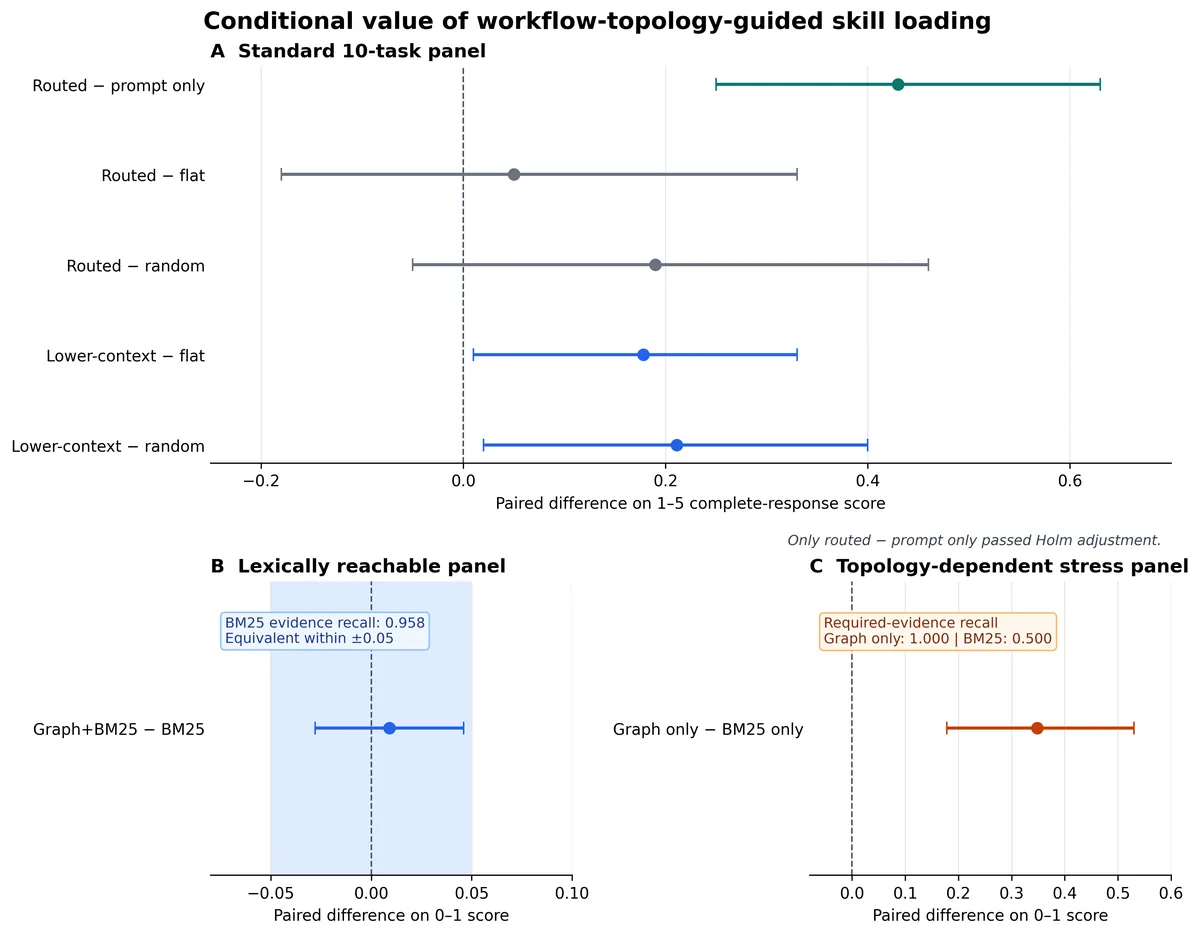
Integrated evidence for the conditional value of workflow topology. (A) Routed-versus-nonrouted contrasts on the 1-5 complete-response rubric in the standard 10-task panel. (B) Graph+BM25 versus BM25-only on the 0-1 deterministic lexically reachable panel, where BM25 evidence recall was already 0.958. (C) Graph-only versus BM25-only on the constructed topology-dependent panel, together with required-evidence recall. Points represent paired task-level mean differences, and whiskers represent paired-task bootstrap 95% CIs. Scales differ by panel, and effects should not be compared numerically across panels.

The lower-context scheduler scored 4.24 versus 4.23 for frozen APS, 4.07 for flat loading, and 4.03 for token-matched random loading. None of the 3 superiority contrasts passed the Holm adjustment. The lower-context-versus-flat difference was 0.18 (95% CI 0.01-0.33; exact *P*=.10; Holm-adjusted *P*=.27). The scheduler used 5684 versus 7986 mean skill-context tokens, a 28.82% reduction, and met the prespecified engineering retention rule. This does not establish cost-effectiveness because the cap deliberately created context headroom, and total integration and review costs were not measured.

Within the context-manifest system, source linkage was present for 559/559 injected fragments, and all 120/120 manifests reproduced exactly. A simulated one-rule change affected an average of 7.56 fewer tasks than flat loading. These were properties of stored context manifests and a simulated change analysis; no human trace-review accuracy, change-control error rate, or operational good practice (GxP) outcomes were measured.

### Boundary Condition Evidence Across Lexical and Topological Regimes

On the lexically reachable 24-task panel, graph+BM25 scored 0.593 and BM25-only scored 0.584 (difference 0.009; 95% CI –0.028 to 0.046; exact and Holm-adjusted *P*=.67; *d*_z_=0.09; Figure 3B; Multimedia Appendix 13). TOST supported equivalence within margins of 0.05 and 0.08, but not 0.03. Both retrieval arms used a mean of 1333 canonical tokens versus 8000 for flat loading.

The evidence audit helped explain the small contrast. BM25-only recalled 95.8% of registry-required sections; graph expansion added no registry-required section; and 21/24 tasks required evidence from one named node. Across arms, 225 criterion failures occurred despite required evidence being present, compared with 16 failures attributable to retrieval. Thus, the panel mainly tested generation after successful retrieval, leaving little opportunity for topology to improve evidence access.

The topology-dependent stress panel deliberately reversed those conditions (Figure 3C; Multimedia Appendix 14). Graph-only scored 0.715 and recalled the required evidence in all tasks, whereas full-corpus BM25 scored 0.367 with recall of 0.500 (Table 3). The paired score difference was 0.348 (95% CI 0.178-0.530; nominal exact *P*=.002; *d*_z_=0.89; post hoc Holm-adjusted *P*=.006 in the sensitivity analysis). Graph-only also exceeded flat loading by 0.626 and random loading by 0.619 (both nominal exact *P*<.001). Typed-edge priority did not improve graph-only selection (–0.052; *P*=.13). Graph+BM25 and BM25-only were similar on the same stress panel (–0.015; *P≥*.99), suggesting that the implemented lexical ranking may have overridden the topology signal.

**Table 3:** Topology-dependent stress-panel results.

Selection policy	Task score (0-1), mean (SD)	Required-evidence recall
Graph-only	0.715 (0.266)	1.000
Graph-only with typed-edge priority	0.663 (0.260)	1.000
Full-corpus BM25^a^	0.367 (0.365)	0.500
Graph expansion+BM25	0.352 (0.311)	0.444
Flat loading at 8000 tokens	0.089 (0.249)	0.111
Token-matched random loading	0.096 (0.140)	0.019

^a^BM25: Best Match 25.

Sensitivity analyses confirmed that the stress-panel result was robust in direction across analysis choices. Collapsing repeated prompts to 14 unique required-evidence targets reduced graph-only minus BM25-only to 0.251 (exact *P*=.02), still a substantial effect. The provenance criterion contributed 40%-50% of each task score; a bounded reconstruction excluding provenance produced an 18-task content-criterion difference of 0.241-0.278 (exact *P*=.03-.047), which attenuated to 0.131-0.143 (*P*=.25) after collapsing repeated targets. Because criterion-level records were not retained, this sensitivity could not be reconstructed exactly. The evidence-routing effect on the constructed panel is clear; the magnitude of the downstream substantive-quality benefit is less certain.

### Evaluator Concordance and Secondary Findings

The practicing programmer and automated judge showed substantial agreement on terminology (quadratic-weighted κ=0.61), moderate agreement on completeness (κ=0.54) and for overall score (intraclass correlation coefficient [ICC(2,1)]=0.60), and insufficient agreement for structure (κ=0.35; Multimedia Appendix 9). The expert scored structure 0.23 points lower on average (95% CI –0.40 to –0.07), consistent with a stricter production-deployability interpretation. The expert reproduced the direction of all 3 component contrasts and judged APS versus domain prompt as the largest (0.37), but no expert contrast passed the Holm adjustment with one output per cell. These results calibrate one judge against one professional; they do not establish expert consensus.

A Define-XML reversal appeared in the persona-panel scores but was not seen in the complete-response reanalysis or the component comparison; we therefore do not interpret metadata synthesis as a stable failure mode of the architecture. Adding the distilled principles store changed scores by –0.05 and –0.02 across the 2 analyses, providing no evidence of incremental benefit on this benchmark.

The recorded prompt-plus-completion cost of the bundled domain-context condition was US $0.182 per run versus US $0.163 for the bare agent. These values omit integration, validation, infrastructure, and human review and are not a cost-effectiveness analysis.

## Discussion

### Principal Findings

The combined experiments yield a clear conditional finding. Curated regulatory context improved automated benchmark scores relative to bare or prompt-only conditions, and bounded scheduling retained quality while reducing injected context by 28.82% with fully reproducible, source-linked manifests. The deterministic panels reveal when topology changes evidence access: on tasks that named the required node and allowed BM25 to recover 95.8% of registered evidence, graph+BM25 and BM25-only were practically similar within a 0.05 margin. On tasks where the prompt lacked the relevant vocabulary but the workflow graph linked the active node to the required source, graph-only selection recovered all required evidence and substantially outscored BM25-only. The graph was therefore most useful precisely when it carried retrieval information not present in the query—a boundary condition with direct implications for task-adaptive retrieval policy. The scope of these findings is bounded by the synthetic setting, a single backbone, and a single expert rater, as detailed in the Limitations.

### Interpretation: Topology Is a Conditional Retrieval Prior

The null and positive contrasts define a single boundary condition rather than conflicting results. In the lexically reachable regime, graph, and BM25 selected nearly the same required evidence. Once evidence was present, most remaining failures were generation errors—omitted fields, derivation mistakes, or schema failures—that retrieval alone could not repair. In the topology-dependent regime, the prompt did not identify the evidence lexically, so graph adjacency supplied new information.

The graph+BM25 arm did not outperform BM25-only even on the stress panel, while graph-only did. This suggests that combining signals is not automatically beneficial. In the implemented pipeline, lexical scoring could rank graph-expanded candidates in a way that diluted the adjacency signal. A future hybrid should use a shared candidate universe and admission policy with orthogonal graph and lexical score terms; the present diagonal comparison changes both selector and context volume and cannot estimate a causal interaction.

This framing also clarifies the value of flat and random controls. Their role is not to represent desirable retrieval methods. They test whether any curated context is sufficient. The component comparison showed that the generator-benchmark-evaluator system was relatively insensitive to which curated files were loaded on the standard 10-task panel. That insensitivity could reflect model parametric knowledge, informative prompts, easy tasks, broad scoring criteria, or judge behavior; it should not be attributed to one cause without a targeted study.

### Context Control and Governance

The lower-context scheduler met the predefined quality-retention criterion while using 28.82% less rule context and reproduced source-linked manifests. Such controls are valuable when reviewers need to know which versioned rules were exposed to the model or which tasks could be affected by a rule change. A manifest records model input, not whether the output used the evidence correctly. The lexically reachable phase reinforced this distinction: provenance-citation criteria passed in 94% of graph-routed runs and 92% of BM25-only runs but in almost none of the flat-loading or random-loading runs, partly because those arms lacked usable evidence identifiers. Manifest and citation outcomes should therefore be interpreted alongside, not as substitutes for, substantive artifact quality.

### Relation to Prior Work

RAG grounds generation in retrieved documents [21,22], and knowledge graphs can represent relations absent from surface text [26]. The present results connect these ideas. The topology was most useful when the relation structure compensated for lexical mismatch. This is compatible with hybrid retrieval but argues against treating graph routing as a universal replacement for lexical search. For routine, explicitly named tasks, a simpler lexical retriever may be sufficient. For multistage tasks whose evidence resides in dependencies not mentioned in the query, topology can supply an additional retrieval prior.

The study differs from general multiagent clinical systems [23-25,33,34] by focusing on bounded rule exposure for regulatory programming artifacts. It also highlights an evaluation issue relevant beyond this domain. A benchmark cannot identify the value of a retrieval signal if its tasks do not require information that signal uniquely provides.

### Practical Implications

The current architecture should be viewed as a research prototype for controlled context assembly. A practical implementation could choose retrieval policy by task regime: lexical retrieval for directly specified work, topology-expanded or topology-prioritized retrieval when dependencies imply evidence beyond the prompt, and a global metadata route for cross-dataset synthesis. Such a policy would require a reliable task classifier and explicit abstention or escalation when the required evidence is uncertain. Experience with clinical AI deployment has shown that benchmark performance does not automatically translate to operational utility [35], underscoring the need for field evaluation before any adoption.

The US Food and Drug Administration’s draft guidance on AI used to support regulatory decision-making and the European Medicines Agency’s reflection paper emphasize validation and human oversight [12,36]. These documents motivate evaluation; they do not certify the present system. Qualified clinical data managers, statistical programmers, and regulatory professionals would remain responsible for artifact validity.

### Limitations

#### Author-Constructed System and Tasks

The graph, 59-file corpus, synthetic fixtures, deterministic criteria, and stress panel were created by one author. The rule files were not independently audited. In the stress panel, task wording, graph relationships, required evidence, and scoring criteria were aligned after an oracle check. This makes the panel useful for demonstrating that the implemented selector can exploit the graph but weak for estimating performance on naturally occurring tasks.

#### Nonfactorial Stress Comparison

Graph-only versus BM25-only changed the graph signal, lexical signal, admission policy, and mean context volume. The contrast is not a main effect graph. Fixed execution order and incomplete response-level artifact retention further limit causal interpretation. The total score also gave 40%-50% weight to a provenance criterion that was mechanically easier to satisfy when the required chunk was retrieved.

#### Evaluation Dependence

Complete-response scoring addressed differential truncation but remained dependent on a single automated judge model within each phase. Both judge model identifiers have since been updated by the provider, so the exact judge builds underlying these scores can be reproducible only from the archived scoring records and recorded system fingerprints, not by requesting the same identifiers. Pairwise judgments showed presentation-order sensitivity. Deterministic criteria avoid LLM-based judging but reflect author-selected criteria and weights. One practicing programmer provided useful concordance evidence, but structure agreement was insufficient, and a single rater cannot establish professional consensus.

#### Synthetic Setting and Model Scope

All results came from synthetic tasks and one GLM-5 backbone. Real sponsor environments contain legacy data, amendments, incomplete metadata, local conventions, security constraints, and iterative human review. Effects may differ across models, context windows, workflow graphs, organizations, and CDISC versions.

#### Statistical Resolution

The first 2 controlled comparisons had 10 task-level observations per contrast despite larger run counts. Several nonsignificant routing contrasts were also not equivalent within margins of 0.15-0.20 and remain unresolved. The stress panel contained 18 task IDs but only 14 unique evidence targets. Post hoc TOST margins, achieved-power diagnostics, and Holm sensitivities aid interpretation but do not create prospective confirmatory inference.

#### Mechanism and Outcome Boundaries

The domain-context phase evaluated node-bound context rather than weighted graph scheduling. No stable Define-XML disadvantage or distilled principles decrement was observed across the complete-response and component analyses. Context reduction was partly enforced by policy. Manifest provenance, reproducibility, and simulated change scope do not establish operational GxP compliance, reduced review time, fewer regulatory queries, or better change-control decisions.

### Future Validation

A decisive next study should be new rather than another increment to the same panel. Tasks and gold evidence should be authored independently of the workflow graph and retrieval outputs, then mapped to a frozen graph after tasks are locked. A shared-pipeline 2×2 design should toggle graph and lexical score terms while holding candidate universe, chunking, selector, token budget, serialization, and tie-breaking constant. Provenance should be a separate mechanism end point; the primary end point should contain independently authored substantive criteria.

The inferential unit should be an independent dependency/evidence family, not a prompt variant, model repetition, or criterion. Execution order should be masked and randomized within complete blocks, with all contexts, responses, criterion records, model identifiers, retries, timestamps, and manifests retained. At least one additional backbone, multiple independently constructed workflow graphs, and multiple independent clinical data management, statistical programming, and regulatory raters are needed.

### Conclusions

This sequential exploratory evaluation identifies a useful boundary condition for workflow topology–guided retrieval. Topology adds little measurable value when lexical retrieval already reaches the required evidence, but it recovers dependency-linked evidence when task wording does not identify it lexically. Curated context improved benchmark scores relative to prompt-only conditions, and bounded scheduling retained quality with substantially less injected context and reproducible manifests. These contributions—a conditional retrieval insight, a context efficiency demonstration, and a governance-aware evaluation framework—provide a concrete foundation for the independently authored factorial validation that the field now needs.

## Appendix

Multimedia Appendix 1 Adapted TRIPOD+AI reporting checklist.

Multimedia Appendix 2 Chronological experiment-refinement and implementation log.

Multimedia Appendix 3 Complete 45-node regulatory workflow graph and edge inventory.

Multimedia Appendix 4 Representative SKILL.md rule file.

Multimedia Appendix 5 Adaptive Priority Scheduler pseudocode and band definitions.

Multimedia Appendix 6 Computational environment and recorded token costs.

Multimedia Appendix 7 Large language model evaluator prompts, personas, and randomization procedure.

Multimedia Appendix 8 Complete-response scoring, task-paired analysis, order sensitivity, and provenance hashes.

Multimedia Appendix 9 Blinded expert-automated-judge concordance study.

Multimedia Appendix 10 Per-task and per-dimension results for the domain-context phase.

Multimedia Appendix 11 Persona-panel scores and interpanel agreement.

Multimedia Appendix 12 Component and context-efficiency comparisons, manifest checks, and reproducibility materials.

Multimedia Appendix 13 Lexically reachable deterministic panel, oracle evidence audit, and error decomposition.

Multimedia Appendix 14 Topology-dependent stress panel, graph-only selection analysis, post hoc multiplicity and target-cluster sensitivities, and reproducibility limitations.

Multimedia Appendix 15 Retained records of generative AI use in manuscript preparation: tool roles and version availability, inventory of retained prompts and session logs with representative records reproduced verbatim, and retention boundaries.

## Abbreviations

ADaM: analysis data model
AI: artificial intelligence
APS: Adaptive Priority Scheduler
BM25: Best Match 25
CDISC: Clinical Data Interchange Standards Consortium
CSP: Constrained Skill Priority
GxP: good practice
ICC: intraclass correlation coefficient
LLM: large language model
RAG: retrieval-augmented generation
SDTM: study data tabulation model
TOST: two one-sided tests
TRIPOD-AI: Transparent Reporting of a Multivariable Prediction Model for Individual Prognosis or Diagnosis—AI

## Data Availability

The eSubmission-Benchmark and CSP-Engine source materials are available in the public project repositories, and the exact evaluated revision is preserved as a versioned archival snapshot with a persistent identifier on Zenodo [37]. The reproducibility package provides task configurations, analysis code, stored summary results, and hashes for the main evidence phases. The topology-dependent stress-panel working-copy files were hash-audited, but the available result bundle does not retain all raw responses, contexts, criterion-level evaluations, returned model identifiers, timestamps, attempts, and full manifests. This retention boundary is disclosed so that the stress result is not represented as a complete immutable provenance chain.
