# Supplementary material for: Workflow Topology–Guided Skill Loading for Agentic AI in Clinical Trial Data Pipelines: Sequential Exploratory Evaluation
Source: JMIR Form Res. 2026 Oct 20;10:e100769. doi: 10.2196/100769 (PMC13732176; doi:10.2196/100769)
Supplement: Supplementary file 1 [file formative_v10i1e100769_app1.pdf]

TRIPOD+AI Reporting Guideline Checklist

Multimedia Appendix 1: TRIPOD+AI Checklist for CSP-Engine Evaluation

This checklist follows the TRIPOD+AI (Transparent Reporting of a multivariable prediction model for Individual Prognosis Or Diagnosis — Artificial Intelligence) extension, used here as an adapted transparency checklist: this study is a sequential exploratory benchmark evaluation of an AI system, not a prediction-model study, and no EQUATOR guideline is an exact match. Items are mapped where applicable and marked N/A otherwise; the checklist documents transparency rather than claiming prediction-model compliance.

Notes

- This study evaluates an AI system for regulatory programming support rather than clinical prediction; some TRIPOD items (eg, calibration, discrimination) are adapted to the evaluation context.

- The CLAIM (Checklist for Artificial Intelligence in Medical Imaging) guideline was considered but deemed less applicable since this study does not involve medical imaging.

- STARE-HI (Statement on Reporting of Evaluation Studies in Health Informatics) principles were consulted for the evaluation methodology design.
