# Supplementary material for: Workflow Topology–Guided Skill Loading for Agentic AI in Clinical Trial Data Pipelines: Sequential Exploratory Evaluation
Source: JMIR Form Res. 2026 Oct 20;10:e100769. doi: 10.2196/100769 (PMC13732176; doi:10.2196/100769)
Supplement: Supplementary file 2 [file formative_v10i1e100769_app10.pdf]

Complete Per-Task Results for the Original Three-Condition Experiment

Complete-response, replicate-averaged task results. Each task-condition cell averages 3 stored runs. Overall is the mean of structure, terminology, and completeness. These data replace the earlier prefix-scored table and are secondary evidence for the bundled intervention, not a graph-routing test.

Task	Layer	Condition	Overall	Structure	Terminology	Completeness
T1: SAP parsing	0	Bare agent	2.670	3.000	2.000	3.000
T1: SAP parsing	0	Bundled framework	3.777	4.333	3.333	3.667
T1: SAP parsing	0	Framework + principles	3.557	4.000	3.333	3.333
T2: Protocol setup	0	Bare agent	2.890	3.000	2.667	3.000
T2: Protocol setup	0	Bundled framework	4.330	5.000	4.000	4.000
T2: Protocol setup	0	Framework + principles	4.110	4.667	3.667	4.000
T3: SDTM DM	2	Bare agent	4.000	4.000	4.000	4.000
T3: SDTM DM	2	Bundled framework	5.000	5.000	5.000	5.000
T3: SDTM DM	2	Framework + principles	4.667	4.667	4.667	4.667
T4: SDTM AE	2	Bare agent	3.890	3.667	4.000	4.000
T4: SDTM AE	2	Bundled framework	4.667	4.667	4.667	4.667
T4: SDTM AE	2	Framework + principles	4.333	4.333	4.333	4.333
T5: P21 validation	3	Bare agent	4.000	4.000	4.000	4.000
T5: P21 validation	3	Bundled framework	4.333	4.333	4.333	4.333
T5: P21 validation	3	Framework + principles	4.000	4.000	4.000	4.000
T6: ADSL	4	Bare agent	3.557	3.667	3.333	3.667
T6: ADSL	4	Bundled framework	4.223	4.333	4.000	4.333
T6: ADSL	4	Framework + principles	4.670	5.000	4.000	5.000
T7: ADAE	4	Bare agent	4.000	4.000	4.000	4.000
T7: ADAE	4	Bundled framework	4.557	4.667	4.333	4.667
T7: ADAE	4	Framework + principles	4.890	5.000	4.667	5.000
T8: TFL demographics	5	Bare agent	3.000	3.000	3.000	3.000
T8: TFL demographics	5	Bundled framework	4.557	5.000	4.000	4.667
T8: TFL demographics	5	Framework + principles	4.890	5.000	4.667	5.000
T9: TFL adverse events	5	Bare agent	3.557	3.667	3.000	4.000
T9: TFL adverse events	5	Bundled framework	4.557	4.667	4.333	4.667
T9: TFL adverse events	5	Framework + principles	4.670	5.000	4.000	5.000
T10: Define.xml	6	Bare agent	4.333	4.333	4.333	4.333
T10: Define.xml	6	Bundled framework	4.667	4.667	4.667	4.667
T10: Define.xml	6	Framework + principles	4.333	4.333	4.333	4.333

Condition summaries

Condition	Overall	Structure	Terminology	Completeness
Bare agent	3.5897	3.6333	3.4333	3.7000
Bundled framework	4.4667	4.6667	4.2667	4.4667
Framework + principles	4.4120	4.6000	4.1667	4.4667

Sources and standalone references

1. Machine-readable source: paper/jmir_ai/analysis_original_full/analysis-summary.json; SHA-256 9b19003d004509eabb12fdee9b7320e15482ffd6d2603065c3b73207427359a4.

2. Inferential method: 3 replicates averaged within each task-condition cell; 10,000-resample paired task bootstrap; exact 2-sided sign-flip test over 10 task differences.
