# Supplementary material for: Workflow Topology–Guided Skill Loading for Agentic AI in Clinical Trial Data Pipelines: Sequential Exploratory Evaluation
Source: JMIR Form Res. 2026 Oct 20;10:e100769. doi: 10.2196/100769 (PMC13732176; doi:10.2196/100769)
Supplement: Supplementary file 3 [file formative_v10i1e100769_app11.pdf]

Automated Compliance-Panel Protocol and Agreement

Standalone supplementary material. Author and institution cover-page identifiers have been removed; the scientific content below is retained from the authoritative source PDF.

1. Panel Protocol

Twoindependentevaluationruns(panels)wereconductedusingDeepSeek(deepseek-chat)prompted to emulate a multidisciplinary expert panel. The model was prompted with distinct personas to evaluate all 10 task pairs in a blinded A/B comparison. Each panel comprised 3 perspectives:

- Clinical Data Manager (CDM) persona: instructed to evaluate with expertise in EDC systems, CDASH/SDTM mapping, data cleaning, CRF design, MedDRA/WHODrug coding, and CDISC controlled terminology governance.
- StatisticalProgrammer(SP)persona: instructedtoevaluatewithexpertiseinSAS/R/Python programming for ADaM/TFL production, CDISC ADaM IG v1.3, macro development, Pinnacle 21 validation, and QC/double-programming.
- RegulatorySpecialist(RS)persona: instructedtoevaluatewithexpertiseinICHE3/E6(R2)/E8/ guidelines, FDA21 CFR Part 11, eCTD Module 5 structure, Define.xml 2.0/2.1 specification, and GxP compliance.

The panel evaluators scored each response on 3 dimensions (structure, completeness, terminology) using a 1–5 integer scale, selected a pairwise winner (A, B, or tie), and flagged safety concerns. Pairs were presented with randomized A/B assignment to prevent position bias, and condition labels (framework vs baseline) were hidden from the evaluating agents. These panels are LLM-based computationalproxiesforexpertjudgment, nothumanexperts; thejudgemodelfamily(DeepSeek) is distinct from the generation backbone (GLM-5).

2. Per-Pair Panel Scores

3. Interpanel and Panel–Judge Agreement

4. Per-Layer Panel Score Differentials

Both panels and the automated LLM judge consistently identified (a) the strongest framework advantage at layer 0 (protocol and planning tasks), (b) moderate advantages across layers 2–5, and (c) a reversal at layer 6, where the baseline outperformed the framework. The persona-conditioned differentialswereuniformlylargerthantheautomatedjudge’s,suggestingthatpersonaconditioning increases evaluator sensitivity to the quality gaps that the framework addresses.

		Panel 1				Panel 2				LLM judge	
Pair	Task	FW	BL	Δ	Choice	FW	BL	Δ	Choice	Δ	Choice
1	ADAE (L4)	5.00	4.00	+1.00	FW	5.00	3.33	+1.67	FW	+1.00	FW
2	SAP (L0)	3.67	2.67	+1.00	FW	4.00	2.33	+1.67	FW	+1.00	FW
3	P21 (L3)	4.33	3.67	+0.67	FW	4.67	3.33	+1.33	FW	+0.67	FW
4	TFL Demo (L5)	3.67	3.00	+0.67	FW	4.00	3.00	+1.00	FW	+0.67	FW
5	ADSL (L4)	4.67	3.67	+1.00	FW	5.00	3.33	+1.67	FW	+1.00	FW
6	TFL AE (L5)	4.67	4.00	+0.67	FW	5.00	4.00	+1.00	FW	+0.67	FW
7	Protocol (L0)	4.33	2.67	+1.67	FW	4.67	2.67	+2.00	FW	+1.67	FW
8	SDTM DM (L2)	4.67	4.67	+0.00	Tie	5.00	4.67	+0.33	FW	+0.00	Tie
9	Define (L6)	2.67	3.67	−1.00	BL	2.33	4.00	−1.67	BL	−1.00	BL
10	SDTM AE (L2)	4.67	4.67	+0.00	Tie	5.00	4.67	+0.33	Tie	+0.00	Tie

Table 1: Panel evaluation scores. Overall scores (mean of structure, completeness, terminology) from the 2 independent persona-conditioned panels compared with the automated LLM judge. FW = framework (graph-constrained); BL = baseline (unconstrained); L = layer. Where panels disagreed on the pairwise choice (pair 8), both choices are reported. The per-task values in Table 2 of the manuscript are the pooled means of the 2 panels shown here.

Comparison	Pairwise κ	Score r
Panel 1 vs LLM judge	1.000	1.000
Panel 2 vs LLM judge	0.756	0.953
Panel 1 vs Panel 2	0.756	0.953

Table 2: Agreement statistics. κ = Cohen kappa for pairwise choice agreement; r = Pearson correlation for continuous scores. Pooled framework win rate across both panels: 75% (15/20 pairings). Pooled panel score delta (FW − BL): +0.75 (framework 4.35 vs baseline 3.60).

Layer	Tasks	Panel Δ	LLM judge Δ	Agreement
0	SAP, Protocol	+1.59	+1.39	Consistent
2	DM, AE	+0.17	+0.00	Consistent
3	P21	+1.00	+0.10	Consistent
4	ADSL, ADAE	+1.34	+0.45	Consistent
5	TFL Demo, TFL AE	+0.84	+0.56	Consistent
6	Define.xml	−1.34	−0.22	Consistent

Table 3: Per-layer panel score differentials (framework − baseline), averaged across both panels. Positive values indicate the framework outperforms the baseline.

Sources and standalone references

1. The panels are persona-conditioned LLM evaluation runs, not human expert panels.

2. The automated-judge layer differentials in the layer table derive from the earlier first-4000-character prefix protocol and are retained for provenance; the complete-response reanalysis (see the manuscript and its statistical appendix) did not reproduce the layer-6 reversal.

3. Provenance note: the per-pair LLM judge column agrees perfectly with Panel 1 (kappa=1.000; r=1.000), consistent with that column reflecting the same underlying evaluation run as Panel 1 rather than an independent third run. Interpanel reliability claims in the manuscript therefore rest solely on the Panel 1 versus Panel 2 comparison (kappa=0.76; r=0.95).

4. DeepSeek-AI. DeepSeek-V3 Technical Report. arXiv:2412.19437. doi:10.48550/arXiv.2412.19437.
