# Supplementary material for: Workflow Topology–Guided Skill Loading for Agentic AI in Clinical Trial Data Pipelines: Sequential Exploratory Evaluation
Source: JMIR Form Res. 2026 Oct 20;10:e100769. doi: 10.2196/100769 (PMC13732176; doi:10.2196/100769)
Supplement: Supplementary file 4 [file formative_v10i1e100769_app12.pdf]

Multimedia Appendix 12: Six-Condition Component Comparison and Context-Efficiency Experiments

Standalone supplementary material. Author and institution cover-page identifiers have been removed; the scientific content below is retained from the authoritative source.

1. Rationale and Design

The original framework-baseline comparison changed the domain system prompt, corpus access, and routing simultaneously. We therefore ran a six-condition component comparison that separated these components while holding the Claude Code command-line harness, GLM-5 backbone, 10-task panel, and replicate structure constant. Each task-condition cell contained 3 independently executed replicates (180 agent runs total; seed 42). All agent calls used isolated working directories.

The 6 conditions were:

1. 1. **Bare agent:** task prompt only; no CDISC system prompt and no SKILL.md context.
2. 2. **Domain prompt only:** CDISC system prompt; no SKILL.md context.
3. 3. **APS:** domain prompt plus graph-routed SKILL.md context under an 8000-token rule-context budget.
4. 4. **APS plus principles:** APS plus the 2 highest-ranked distilled principles.
5. 5. **Budget-capped flat loading:** all 59 SKILL.md files were offered with uniform priority and deterministic alphabetical ordering. The 8000-token budget admitted 7 files; this is not simultaneous loading of all 59 full files.
6. 6. **Random loading (k=5):** 5 SKILL.md files were reproducibly sampled per task and replicate. This matched the mean skill count approximately, not the skill-context token volume.

2. Complete-Response Scoring

The initial scalar and pairwise judge paths received only the first 4000 characters of each answer. Because 158 of 180 stored responses exceeded that length, the prefix scores were retained for provenance but were not used as final component-comparison outcomes. We scored all complete stored responses with a pinned deepseek-v4-flash nonthinking JSON protocol (temperature 0). The returned model was deepseek-v4-flash for every successful call, with system fingerprint fp_8b330d02d0_prod0820_fp8_kvcache_20260402.

For pairwise sensitivity analysis, each of 90 replicate-matched comparisons was presented in both AB and BA order (180 presentations). A logical pair was counted as an APS win, comparator win, or tie only when both orientations normalized to the same winner; otherwise it was labeled order-discordant. Pairwise results were not used for confirmatory inference.

3. Statistical Analysis

The task was the inferential unit. The 3 replicate scores in each task-condition cell were averaged before paired inference, yielding 10 paired task differences per contrast. We computed 10,000-resample percentile paired-bootstrap 95% confidence intervals, exact 2-sided sign-flip P values, and paired standardized mean differences (dz). Holm adjustment controlled the family-wise error rate across 3 prespecified routing contrasts: APS versus domain prompt only, budget-capped flat loading, and random loading. Four component contrasts were exploratory; a Holm-4 adjustment is shown as a sensitivity analysis.

4. Results

Table 1. Replicate-averaged task-level overall scores.

Condition	Tasks	Mean	SD	Skills / skill tokens
Bare agent	10	3.98	0.57	0.0 / 0
Domain prompt only	10	4.20	0.46	0.0 / 0
Random loading (k=5)	10	4.44	0.45	5.0 / 6768
Budget-capped flat loading	10	4.58	0.29	7.0 / 7986
APS	10	4.63	0.34	4.9 / 7991
APS plus principles	10	4.61	0.30	4.6 / 7731

SD is the standard deviation across the 10 task means. Skill tokens are deterministic injected-context counts; API input-token fields were not used for routing-efficiency claims because cache accounting was incomplete.

Table 2. Prespecified routing contrasts.

Contrast (A-B)	Delta	95% CI	Exact P	Holm P	dz
APS - domain prompt only	+0.43	[0.25, 0.63]	.004	.012	1.30
APS - flat loading	+0.05	[-0.18, 0.33]	.781	.781	0.13
APS - random loading	+0.19	[-0.05, 0.46]	.211	.422	0.43

The routing-superiority gate was not met because APS did not outperform either nonrouted loading strategy.

Table 3. Exploratory component contrasts.

Contrast (A-B)	Delta	95% CI	Exact P	Holm-4 P	dz
Domain prompt only - bare agent	+0.22	[-0.03, 0.46]	.129	.258	0.52
Flat loading - domain	+0.38	[0.14, 0.64]	.018	.070	0.88

prompt only					
Random loading - domain prompt only	+0.24	[0.08, 0.40]	.031	.094	0.87
APS plus principles - APS	-0.02	[-0.11, 0.08]	.828	.828	-0.13

The flat and random comparisons did not remain below .05 after the Holm-4 sensitivity adjustment, and distilled principles provided no detectable benefit.

5. Pairwise Presentation-Order Sensitivity

The pairwise judge showed a strong first-position tendency. It selected the first-presented response in 148 of 180 presentations. Fifty-eight of 90 logical pairs (64%) were order-discordant: the selected condition changed when AB order was reversed to BA. The discordant counts were 21/30 for APS versus domain prompt only, 16/30 for APS versus flat loading, and 21/30 for APS versus random loading. These results are a judge-bias diagnostic and do not corroborate the scalar findings.

6. Interpretation Boundaries

APS improved complete-response scores relative to domain prompt only, but it did not outperform budget-capped flat or random loading. The experiment therefore does not support an outcome-quality advantage from topology-guided routing. The data are compatible with a curated-context effect, but the nonrouted component contrasts were exploratory and did not survive the Holm-4 sensitivity adjustment; "domain priming" remains a hypothesis rather than a demonstrated mechanism.

APS and flat loading consumed essentially the same deterministic skill-context budget (7991 vs 7986 tokens), so the experiment does not support a context-token-savings claim for routing versus flat loading. APS selected fewer files, and its graph-linked selection records are inspectable, but governance or auditability outcomes were not empirically measured. The result is bounded by the 59-file corpus, 10 purposively selected synthetic tasks, one GLM-5 backbone, one automated judge version, the budget-capped alphabetical flat implementation, and a random arm that was not token-matched.

7. Reproducibility and Provenance

The immutable source checkpoint is experiments/results/experiment_v5_corrected_20260717.json (SHA-256 e22b3c2222e810ecd28bb6c032d629bdcbfd089147c0178bd99724aa86a47d59). The complete-response checkpoint is experiments/results/experiment_v5_rejudged_full_outputs.json (analysis source SHA-256 e337862a974fd2bb5cd1bd691875f1e6483b8acca5687cbe4e758f5fcb587ed8). The locked scoring protocol SHA-256 is fcd73a0c78b9b37d66f073eb18c88a2d2d8df5c0328229f8020110687739fb58.

The runner, full-response judge, task-paired analysis, and bundle generator are experiments/run_experiment_v5_corrected.py, experiments/rejudge_v5_full_outputs.py, experiments/analyze_corrected_experiment.py, and experiments/generate_v5_analysis_bundle.py. The cumulative recorded cost was US $29.93, including US $0.21 for complete-response scalar and

bidirectional pairwise judging. Code and evaluation materials are available at https://github.com/yanmingyu92/csp-workflow-engine.

8. Prespecified Context-Efficiency Optimization Follow-Up

After the component comparison and before inspecting any optimization outcomes, we fixed a compact four-arm design using the same 10 tasks, GLM-5 backbone, isolated execution harness, and 3-replicate structure (120 new agent runs total; seed 42):

7. 1. **Frozen APS:** exact APS context selection from the component comparison, augmented only with the provenance manifest.
8. 2. **Optimized APS:** all CURRENT content protected in full; non-CURRENT candidates admitted by deterministic task-level lexical relevance; an 84% soft context cap with an override for full CURRENT demand, bounded by the common 8000-token hard ceiling.
9. 3. **Flat loading:** all 59 files offered with uniform alphabetical priority under the 8000-token ceiling.
10. 4. **Token-matched random loading:** seeded random corpus ordering matched to the realized optimized context volume within 1 estimated token.

The 84% soft cap encoded a 16% context-headroom policy, one percentage point beyond the prespecified 15% reduction gate to accommodate deterministic estimator and truncation rounding. The optimization policy therefore partly enforced the context reduction; preservation of complete-response quality remained empirical. The dated design, A/B/C interpretation rules, and source hashes were fixed before the formal run.

9. Follow-Up Judging and Statistical Analysis

A pinned deepseek-v4-pro judge scored each complete stored response at temperature 0 under the same three-dimension rubric. The returned model was deepseek-v4-pro with system fingerprint fp_9954b31ca7_prod0820_fp8_kvcache_20260402. Pairwise judging was omitted. The locked 2000-token judge output ceiling produced parse failures for some calls: the first pass ended with 26 invalid rows, and checkpointed retries accumulated 58 preserved parse-error attempt records. Only invalid rows were retried under the identical frozen configuration; no valid score was replaced. The final checkpoint contained 120/120 valid full-response judgments with response byte, character, and SHA-256 proof and zero final integrity errors.

The task remained the inferential unit. Replicate scores were averaged within each task-arm cell before analysis. Each of 3 prespecified contrasts used a 10,000-resample paired-task bootstrap 95% CI, an exact 2-sided sign-flip test, and paired dz; Holm adjustment covered optimized APS versus frozen APS, flat loading, and token-matched random loading. Superiority required a positive mean difference and Holm-adjusted $P<.05$. The engineering quality-retention gate required the optimized-minus-flat CI lower bound to be at least -0.20 points; this threshold is not a validated clinical noninferiority margin.

10. Follow-Up Results

Table 4. Replicate-averaged task-level quality in the optimization follow-up.

Arm	Tasks	Mean quality	SD
Optimized APS	10	4.24	0.44
Frozen APS	10	4.23	0.52
Flat loading	10	4.07	0.60
Token-matched random loading	10	4.03	0.50

Table 5. Prespecified optimization contrasts.

Contrast (A-B)	Delta	95% CI	Exact P	Holm P	dz
Optimized - frozen APS	+0.01	[-0.14, 0.17]	1.000	1.000	0.04
Optimized - flat	+0.18	[0.01, 0.33]	.102	.270	0.63
Optimized - token-matched random	+0.21	[0.02, 0.40]	.090	.270	0.66

No contrast met the Holm-adjusted superiority criterion. Positive bootstrap intervals versus flat and random loading are reported as uncertainty estimates and do not override the locked exact-test decision rule.

Table 6. Prespecified engineering and governance gates.

Measure	Observed	Gate
Mean skill-context tokens, optimized / flat	5684 / 7986	Descriptive
Optimized-versus-flat context reduction	28.82%	>=15%: pass
Optimized-minus-flat quality CI lower bound	+0.011	>=-0.20: pass
Provenance completeness	100%	100%: pass
Repeated-manifest reproducibility	100%	100%: pass
Mean affected-task difference, optimized - flat	-7.56	<=0: pass

Provenance, manifest, and simulated change-impact measurements are deterministic architectural proxies; they do not establish operational GxP compliance.

The follow-up therefore met Outcome B: similar quality with an efficiency/governance advantage. It did not meet Outcome A because no superiority contrast passed Holm adjustment. The evidence supports positioning relevance-aware routing as controlled, traceable context management, not as a proven outcome-quality improvement mechanism.

11. Follow-Up Reproducibility and Interpretation Boundaries

The final judged source checkpoint and its validated archival copy are byte-identical at SHA-256 fb9f787a6e95e2bd0813c7f7b90fd986e64adeba09305645572a3875df927aa0. The locked design SHA-256 is 4bc55199fef7c37a8d38cf504aff19529a77ed6ac1b0be7917ba7ba7a95e715b; the runner, optimized-context implementation, and analyzer SHA-256 values are 304238a34de570f6dcb1d920d68eb25d2e0e3009848a21571dc5e77cac82a232,

452cd46ff7d1cb6b067fc54e28314d9deab33e2b7154512603d94ba9e947f617, and 56e08f177aed773b845c3200a6a38a664fbf83bd0c8db0693d3076652592336d. Independent deterministic recomputation reproduced the analysis JSON, report, statistical appendix, figure catalog, and figures exactly.

The optimization runner, context/provenance implementation, and analyzer are experiments/run_experiment_v6_aps_gxp.py, experiments/aps_gxp_context.py, and experiments/analyze_aps_gxp_experiment.py. The formal checkpoint is experiments/results/experiment_v6_aps_gxp_formal_20260724_judged.json. Results are bounded by the 59-file corpus, 10 synthetic tasks, one GLM-5 generator, one DeepSeek judge version, and deterministic engineering proxies. They do not demonstrate equivalence, operational audit performance, human-review savings, real-world change-control accuracy, or GxP compliance.
