# Supplementary material for: Workflow Topology–Guided Skill Loading for Agentic AI in Clinical Trial Data Pipelines: Sequential Exploratory Evaluation
Source: JMIR Form Res. 2026 Oct 20;10:e100769. doi: 10.2196/100769 (PMC13732176; doi:10.2196/100769)
Supplement: Supplementary file 5 [file formative_v10i1e100769_app13.pdf]

Routing-Sensitive Deterministic Follow-Up and Oracle Decomposition

Standalone supplementary material. Author and institution cover-page identifiers have been removed; the scientific content below is retained from the authoritative source PDF.

Note on the workflow-graph edge inventory: the edge counts reported in this appendix describe the audited corpus graph used by the routing-sensitive follow-up, which was revised from the original framework graph inventoried in Multimedia Appendix 3; the two inventories are therefore not expected to match.

Integrity qualification and exact primary result. Graph-routed (graph plus BM25)

0.5926422 versus BM25-only 0.5839120; difference +0.0087302, 95% CI [-0.0281085, 0.0459656], exact and Holm P=.671875; 6 positive, 14 tied, and 4 negative task differences. Graph superiority was not established. The design and decision rule were internally prespecified and hash-recorded. A post-run audit identified 2 corpus-file differences between the design-freeze manifest and the files consistently recorded by all 288 generation manifests; the exact executed corpus snapshot and the drift are documented in the reproducibility release, and no freeze-conformance claim is made.

1. Rationale and design

The component and optimization experiments loaded whole SKILL.md files onto the same 10-task battery and used LLM judging as the outcome instrument. This final prespecified follow-up was designed to (a) maximize sensitivity to retrieval differences by operating at section-level chunk granularity with exactly matched canonical token budgets, (b) replace the subjective outcome with machine-verifiable deterministic criteria, and (c) evaluate genuinely held-out tasks. It was executed on the audited execution snapshot of the skill corpus in which core sections received

explicit provenance and version labels; scores from this follow-up are therefore not comparable to the means of the earlier experiments, and no cross-experiment mean comparison is made.

Twenty-fournewheld-outtaskswereinternallyspecifiedwithmachine-verifiableatomiccriteria coveringexactvariables,controlled-terminologyandcodelistversions,forbiddenhallucinatedterms, derivation formulas, XML/JSON/YAML schema validity, executable code where feasible, provenance citations, and upstream change propagation. Four arms were compared, all under exactly matched canonical context-token budgets except the flat ceiling arm:

1. Graph-routed retrieval: dependency-aware graph expansion (CURRENT-node service floor of 320 tokens; controlled 2-hop penalty 0.5; score-gap ratio 0.35; maximal-marginal-relevanceλ = 0.68)combinedwithBM25(BestMatch25)lexicalchunkscoring,withversion-conflict exclusion and deterministic provenance manifests.

2. Full-corpus BM25-only retrieval: BM25chunkscoringoverthefullcorpuswithnograph information, at the exact canonical token budget of arm 1.
3. Random retrieval: deterministic seeded random chunk selection at the same exact token budget.

4. Flat loading: flat context at the 8000-token ceiling.

Threereplicateswereexecutedforeverytask-armcell(24tasks×4arms×3replicates; N=288 runs; GLM-5 backbone via the Claude Code harness; isolated working directories; fail-closed checkpoint/resume). Router parameters were selected exclusively from retrieval diagnostics on 10 development tasks disjoint from the held-out set (maximum pairwise prompt similarity 0.20); no generation or judge score was available during tuning. Task definitions, atomic criteria, validators, arm configuration, prompts, seeds, router configuration, and analysis code were internally prespecified and hash-recorded before the first paid call; the 2-file corpus drift identified by the post-run audit is documented in the reproducibility release. The internally prespecified decision rule permitted a routing-superiority claim only if the graph-routed arm exceeded both the BM25-only and random arms after Holm adjustment.

2. Endpoints and statistical analysis

The primary endpoint was the deterministic criterion score: each repetition's earned atomic-criterion weight divided by available weight (0–1 scale), under a frozen missing/failure policy in which missing, unparseable, schema-invalid, or validator-crashing outputs scored zero for the affected criteria. A blinded deepseek-v4-pro readability/actionability score over complete stored responses was secondary; the judge received no arm identity or retrieval metadata. The task was the inferential unit: 3 replicates were averaged within each task-arm cell, yielding 24 paired task differences per contrast. We computed 20,000-resample percentile paired-bootstrap 95% confidence intervals,exact2-sidedsign-flipP values,pairedstandardizedmeandifferences(d_z),leave-one-task-out (LOO) ranges, and Holm adjustment across the 2 prespecified routing contrasts (graph-routed vs BM25-only; graph-routed vs random); the graph-routed vs flat contrast was supporting, with a prespecified retention rule (paired bootstrap lower bound ≥ -0.05 on the 0–1 scale plus a positive canonical context-token reduction).

3. Primary and secondary results

Arm	Deterministic mean (0–1)	Judge mean (1–5)	Context tokens
Graph-routed retrieval	0.5926	4.86	1333
Full-corpus BM25-only	0.5839	4.86	1333
Random retrieval	0.2268	4.05	1333
Flat loading (8000)	0.2227	4.22	8000

Table 1: Replicate-averaged task-level means over the 24 held-out tasks. Canonical context tokens are means of the deterministic tokenizer counts recorded in the context manifests.

A sensitivity analysis removing the provenance-citation criterion weight reduced the primary contrast to +0.0005 (95% CI [−0.0493,0.0491]), indicating that almost all of the small graph-vs-BM25pointdifferencewascarriedbytheexplicitevidence-citationcriterionratherthansubstantive artifact content.

Contrast (A–B)	Δ	95% CI	Exact P	Holm P	d_z
Graph-routed − BM25-only	+0.0087	[−0.0281,0.0460]	.6719	.6719	0.09
Graph-routed − random	+0.3658	[0.2908,0.4371]	3.6×10^{-7}	7.2×10^{-7}	1.95
Graph-routed − flat (supporting)	+0.3699	[0.2853,0.4519]	4.8×10^{-7}	—	1.74

Table 2: Task-paired deterministic contrasts (24 paired tasks; 20,000-resample paired bootstrap; exact 2-sided sign-flip tests; Holm adjustment over the 2 prespecified routing contrasts). For the primary graph-vs-BM25 contrast, 6 task differences were positive, 4 negative, and 14 exact ties; the LOO mean range was [−0.0012,0.0188] and the LOO sign was not stable. The internally prespecified routing-superiority rule was not met. The retention rule versus flat passed with an 83.34% canonical context reduction (1333 vs 8000 tokens).

4. Governance evidence audit

All 288 context manifests passed schema and repeated-hash checks; provider cache fields and serialized prompt counts were complete; 144 excluded version conflicts were detected with zero stale chunks selected; and task-aggregated deterministic change containment passed. Response-level provenance-citation criteria passed in 46.9% of cells pooled across all 4 arms: 94% in the graph-routed arm and 92% in the BM25-only arm, but near 0% in the random and flat arms, which received no usable manifest evidence identifiers. The pooled figure therefore does not support a broad provenance-quality claim; the per-arm figures show that citation-grounded outputs required manifest-based retrieval. These are benchmark-specific architectural measurements, not evidence of operational GxP compliance.

5. Offline oracle retrieval and error decomposition

After the formal analysis was frozen, a fully offline, deterministic oracle audit reconstructed all 288 cells and 1236 criterion records from stored manifests, complete responses, and criterion histories (no model generation or judging was run). Two independent reruns were byte-identical, and an independentverifierconfirmed8frozensourcefiles,59corpusfiles,and288generation-to-evaluation linkages with zero mismatches.

Oracle metric	Graph-routed	BM25-only
Required-section recall	0.9583	0.9583
Required-section precision	0.2177	0.2196
Required-token precision	0.2532	0.2570
Dependency coverage	0.9583	0.9583
Irrelevant-token ratio	0.7468	0.7430
Redundancy	0.1191	0.1131
Version-selection correctness	1.0000	1.0000
Manifest provenance completeness	1.0000	1.0000

Table 3: Oracle retrieval metrics against the frozen task-to-required-evidence registry. BM25-only retrieval reached perfect required-section recall on 23 of 24 tasks. Across 72 paired task-replicate comparisons, graph expansion added 57 chunks not present in the matched BM25 context, of which 0 were registry-required; the graph therefore did not supplement any BM25-missed required section on this panel.

Primary error class (failed criteria)	Graph-routed	BM25-only
Retrieval miss	8	8
Wrong or stale evidence	0	0
Evidence present, generator omission	33	33
Derivation error	58	61
Schema or serialization error	12	12
Forbidden hallucination	5	7
Citation mismatch	1	3
Judge-only disagreement	1	3

Table 4: Mutually exclusive primary error classes for failed atomic criteria in the 2 retrieval arms. Retrieval absence was evaluated before generator attribution. Evidence-present output failures totaled 225 versus 16 retrieval-side failures, indicating a downstream generation bottleneck under the frozen evidence registry.

Three structural findings contextualize the null routing contrast. First, 21 of 24 tasks required evidence from only a single named workflow node (3 required multi-hop evidence), so the panel offered little identifying variation for topology-guided retrieval. Second, no cell occupied the evidence-improved stratum: required-section recall was identical in every graph/BM25 pair, so the follow-up cannot estimate whether output improves when graph retrieval uniquely repairs a lexical-retrieval miss, and no graph-only arm was included. Third, the router treated workflow adjacency as symmetric evidence dependency and did not use typed edge semantics (edge inventory: requires 80, enables 15, validates 3) in its dependency distance; development-stage retrieval precision (0.3483) also shifted downward on held-out tasks (0.2177). Exploratory, non-multiplicity-adjusted strata showed a more favorable graph point difference on low lexical-overlap tasks (+0.059; 8 tasks) thanonhigh-overlaptasks(−0.045; 8tasks); thesearediagnosticassociationsonly. Whethertyped dependency traversal helps when required evidence is lexically mismatched or distributed across nodes remains an open question for an internally prespecified graph-on/off by retrieval-on/off design with oracle evidence gain as the primary endpoint.

6. Reproducibility provenance

Hash-recorded artifact	SHA-256
Pre-run configuration freeze	fe5ba9580ceb7096a89bcb29e871cde68d27a3e446e26f371f4fb4ec8fed1880
Formal generation checkpoint	f53150fb9bf6a46817dbb1fd0ba2f53f8f78ed73e84ac3aa1ae61148f8a3f6d2
Formal evaluated results	98c452dbf72eca1c917de093fccaedcd664aa2a2aef6f99dc95c6ca6424e3837
Failure log	aa9bfe4cec2edb6db21720beeea38ab704274fdb841a3ff0652a4b544b361972
Statistical analysis output	87e9d6959fa1c7aa0d1bcce0f4f76ee4f52ccab9a82a7e888c3cebbcfd133127
Governance audit content	734d38a91ce85cf1850591b4f9d16f1e8492564ec220668717de99b8c5d549db

Table 5: SHA-256 hashes of the frozen routing-sensitive follow-up artifacts. Four transient generation attempts failed and were retained before successful fail-closed retries; there were no missing cells, judge failures, or silent substitutions. A second seeded analysis run reproduced the analysis output byte-for-byte. The immutable optimization-experiment checkpoint (Multimedia Appendix 12) was preserved unchanged throughout.

All artifacts, runners, validators, the frozen task registry, and the oracle decomposition code are included in the versioned reproducibility release referenced in the Data Availability statement (doi:10.5281/zenodo.21614975).

7. Standalone notes

1. Primaryresultsource: experiments/results/experiment_v7_routing_sensitive_formal_evaluated_20260725.json

2. Asubsequentofflinetyped-graphretrievalprototype hasstatuspost hoc retrieval only not confirmatory; it is not evidence of graph superiority and is not part of this study’s claims.

3. The reproducibility release distinguishes the design-freeze hashes from the exact executed corpus snapshot and documents the 2-file drift.

Sources and standalone references

1. Panel result source: experiments/results/experiment_v7_routing_sensitive_formal_evaluated_20260725.json.
2. V8 status is post_hoc_retrieval_only_not_confirmatory; it is not evidence of graph superiority.
3. The release must distinguish design-freeze hashes from the exact executed corpus and document the 2-file drift.
