# Supplementary material for: Workflow Topology–Guided Skill Loading for Agentic AI in Clinical Trial Data Pipelines: Sequential Exploratory Evaluation
Source: JMIR Form Res. 2026 Oct 20;10:e100769. doi: 10.2196/100769 (PMC13732176; doi:10.2196/100769)
Supplement: Supplementary file 6 [file formative_v10i1e100769_app2.pdf]

Multimedia Appendix 2: Sequential Experiment Implementation Log

Purpose

This appendix documents the implementation details of each experimental phase for reproducibility. The main manuscript organizes the evidence by scientific regime rather than chronologically. None of the phases was publicly preregistered.

Original Bundled Comparison and Complete-Response Scoring

The original comparison executed 90 runs (10 tasks x 3 conditions x 3 replicates). The initial judge path evaluated only the first 4000 characters. Complete stored responses were scored under a locked full-response protocol. The bundled domain-prompt-plus-node-bound-rule condition scored 4.4667 versus 3.5897 for the bare agent (difference +0.8770; 95% CI 0.6333-1.1320; exact P=.001953). Because prompt and rule access changed together and the scheduler was not applied in this phase, this is bundled-intervention evidence, not a routing test.

Component Comparison Phase

The component comparison executed 180 runs (10 tasks x 6 arms x 3 replicates). APS scored 4.6333 versus 4.2007 for domain prompt only (difference +0.4327; 95% CI 0.245-0.632; exact P=.003906; Holm P=.011719). APS did not outperform flat or random curated-context loading. This supports a curated-context contrast on the standard panel but does not isolate routing as the source.

Context-Efficiency and Manifest Phase

The context-efficiency follow-up executed 120 runs (10 tasks x 4 arms x 3 replicates). Optimized APS scored 4.2444, flat loading 4.0667, and token-matched random loading 4.0333; no superiority contrast passed Holm adjustment. Optimized APS used 28.8217% less injected skill context than flat loading. Within the manifest system, 559/559 injected fragments had recorded sources and 120/120 manifests reproduced exactly.

Lexically Reachable Deterministic Panel

The lexically reachable panel executed 288 runs (24 held-out tasks x 4 arms x 3 replicates). Graph+BM25 scored 0.5926422 and full-corpus BM25 scored 0.5839120 (difference +0.0087302; 95% CI -0.0281085 to 0.0459656; exact and Holm P=.671875). Graph superiority was not established. BM25 recalled 23/24 registered required sections, graph expansion added none, and evidence-present generation failures dominated retrieval failures. The panel therefore had little opportunity to identify incremental topology value.

A post-run comparison identified 2 corpus-file differences from the design-freeze manifest. The reproducibility release preserves the original freeze artifact, supplies the exact executed corpus snapshot separately, and documents the drift; it does not claim freeze conformance.

Post Hoc Retrieval Diagnostic

An offline typed-graph retrieval diagnostic was conducted after the formal panels. Its status is labeled post hoc retrieval only, not confirmatory. The diagnostics informed later design but are not evidence of graph superiority.

Topology-Dependent Mechanism Stress Panel

The topology-dependent stress panel executed 324 runs (18 author-constructed tasks x 6 arms x 3 replicates) with required evidence deliberately dependency-linked and lexically mismatched with task prompts. Graph-only selection scored 0.715 with required-evidence recall 1.000; full-corpus BM25 scored 0.367 with recall 0.500. The paired difference was +0.348 (95% CI 0.178-0.530; nominal exact P=.001953; paired dz=0.893).

This is a mechanism demonstration under fixed constructed conditions, not a clean factorial graph effect. Selector/admission policy and context volume differed, 18 task IDs represented 14 unique evidence targets, provenance contributed 40%-50% of the score, and criterion-level response records were not retained. Target-collapsed and provenance-excluding sensitivities bound the supported conclusion to evidence-access improvement rather than general downstream quality superiority.

Evidence-Chain Summary

Phase	Runs/tasks	Principal result	Supported decision
Component comparison	180/10	APS-prompt +0.4327; Holm P=.011719	Positive curated-context contrast; routing not isolated
Context efficiency	120/10	Optimized-flat +0.1778; Holm P=.2695	No superiority; context-retention and manifest evidence
Lexically reachable panel	288/24	Graph+BM25-BM25 +0.0087; Holm P=.671875	No incremental topology value on lexically reachable panel
Post hoc retrieval diagnostic	Offline	Typed-graph retrieval diagnostics	Post hoc; not confirmatory
Topology-dependent stress panel	324/18	Graph-only-BM25 +0.3481; nominal P=.001953	Conditional mechanism evidence; nonfactorial

Primary Machine-Readable Sources

- experiments/results/experiment_v5_rejudged_full_outputs.json
- experiments/results/experiment_v6_aps_gxp_formal_20260724_judged.json

- experiments/results/experiment_v7_routing_sensitive_formal_evaluated_20260725.json
- experiments/results/experiment_v8_typed_graph_retrieval_20260725/analysis.json
- experiments/results/v9_graph_only_live_analysis.json
- experiments/config/experiment_v9_routing_hostile_tasks.yaml
