# Supplementary material for: Workflow Topology–Guided Skill Loading for Agentic AI in Clinical Trial Data Pipelines: Sequential Exploratory Evaluation
Source: JMIR Form Res. 2026 Oct 20;10:e100769. doi: 10.2196/100769 (PMC13732176; doi:10.2196/100769)
Supplement: Supplementary file 7 [file formative_v10i1e100769_app3.pdf]

Complete Regulatory Workflow Graph Node Inventory

Standalone inventory of the 45-node, 7-layer directed acyclic graph documented for the original framework. Requires denotes a hard dependency, Enables a soft dependency, and Validates a cross-check dependency.

Node	Layer	Task description	Recorded edge relationships
1	0	Protocol parsing	Enables -> 4, 5
2	0	SAP section extraction	Requires -> 1
3	0	Endpoint identification	Requires -> 2
4	0	Visit schedule mapping	Enables -> 9
5	0	Inclusion/exclusion criteria	Enables -> 9
6	0	Treatment arm specification	Requires -> 3
7	0	SAP-derived variable list	Requires -> 2; Validates -> 15
8	0	Analysis set definitions	Requires -> 7
9	1	Demographics (DM) import	Enables -> 16
10	1	AE raw data import	Enables -> 17
11	1	Concomitant medications import	Enables -> 18
12	1	Laboratory data import	Enables -> 19
13	1	Vital signs import	Enables -> 20
14	1	Exposure (EX) import	Enables -> 21
15	1	Subject disposition import	Validates -> 9
16	2	DM domain mapping	Requires -> 9
17	2	AE domain mapping	Requires -> 10
18	2	CM domain mapping	Requires -> 11
19	2	LB domain mapping	Requires -> 12
20	2	VS domain mapping	Requires -> 13
21	2	EX domain mapping	Requires -> 14
22	2	DS domain mapping	Requires -> 15
23	2	Supplemental qualifiers	Validates -> 16-22
24	3	Cross-domain consistency	Validates -> 16-22
25	3	Controlled terminology check	Requires -> 23
26	3	Completion status validation	Validates -> 24
27	3	Date/logic checks	Validates -> 16-22
28	3	Pinnacle 21 compliance	Requires -> 24-27
29	4	ADSL derivation	Requires -> 16, 22
30	4	ADAE derivation	Requires -> 17, 29
31	4	ADCM derivation	Requires -> 18, 29
32	4	ADLB derivation	Requires -> 19, 29
33	4	ADVS derivation	Requires -> 20, 29
34	4	ADEX derivation	Requires -> 21, 29
35	4	Parameter derivation logic	Requires -> 29
36	4	Flag variable computation	Requires -> 35
37	4	ADaM metadata compliance	Validates -> 29-36
38	5	Statistical table generation	Requires -> 29-37
39	5	Listing generation	Requires -> 29-37
40	5	Figure generation	Requires -> 29-37
41	5	QC/reproduction	Validates -> 38-40

Node	Layer	Task description	Recorded edge relationships
42	5	Analysis review package	Requires -> 41
43	6	Define.xml generation	Requires -> 23, 28, 37
44	6	Reviewer's guide compilation	Requires -> 43
45	6	eCTD packaging	Requires -> 43, 44

Sources and standalone references

1. The complete machine-readable graph and corpus are included in the versioned reproducibility release (doi:10.5281/zenodo.21614975).

2. Pan S, Luo L, Wang Y, et al. Unifying large language models and knowledge graphs: a roadmap. IEEE Trans Knowl Data Eng. 2024;36(7):3580-3599. doi:10.1109/TKDE.2024.3352100.
