# Supplementary material for: Workflow Topology–Guided Skill Loading for Agentic AI in Clinical Trial Data Pipelines: Sequential Exploratory Evaluation
Source: JMIR Form Res. 2026 Oct 20;10:e100769. doi: 10.2196/100769 (PMC13732176; doi:10.2196/100769)
Supplement: Supplementary file 8 [file formative_v10i1e100769_app4.pdf]

Representative SKILL.md Rule File

Abridged excerpt of the adverse-event mapping rule file (sdtm-ae-mapper) bound to Node 17. The complete file, including the MedDRA coding hierarchy, edge-case handling, and audited derivation constraints, is included in the versioned reproducibility release. This example illustrates rule-file structure; it is not a CDISC certification or a substitute for the applicable implementation guide.

```
# SDTM AE Domain Mapping (sdtm-ae-mapper) - abridged excerpt

## Objective
Map raw adverse event data to the SDTM AE domain with MedDRA coding,
following CDISC SDTMIG v3.4.

## Variable Specification (excerpt)
| Variable | Required | Source  | Notes                                      |
|----------|----------|---------|--------------------------------------------|
| STUDYID  | Yes      | Config  | Study identifier                           |
| DOMAIN   | Yes      | -       | Fixed value: "AE"                          |
| USUBJID  | Yes      | Derived | {study_id}-{SITEID}-{SUBJID}; must match DM |
| AESEQ    | Yes      | Derived | Sequence number within subject             |
| AETERM   | Yes      | RAW.AE  | Verbatim adverse event term                |
| AEDECOD  | Yes      | MedDRA  | MedDRA preferred term (dictionary-derived) |
| AEBODSYS | Yes      | MedDRA  | MedDRA primary system organ class          |
| AESEV    | Yes      | RAW.AE  | Severity mapped to CDISC CT C66769         |
| AESER    | Yes      | Derived | From individual seriousness criteria       |
| AESTDTC  | Yes      | RAW.AE  | Start date/time (ISO 8601)                 |
| AEENDTC  | No       | RAW.AE  | End date/time (ISO 8601)                   |

## Controlled Terminology (excerpt)
- AESEV (CDISC CT C66769): MILD | MODERATE | SEVERE
- AESER (CDISC CT C66763): Y | N, derived from the individual seriousness
  criteria AESDTH, AESLIFE, AESHOSP, AESDISAB, AESCONG, and AESMIE

## Mapping Rules (excerpt)
1. AETERM must match the source verbatim; never modify verbatim terms.
2. AEDECOD requires MedDRA coding with the study-pinned dictionary version,
   documented for every run.
3. Dates must follow ISO 8601; preserve partial dates without imputation.
4. Validate USUBJID against the DM domain.

## Safety Constraints (excerpt)
- NEVER invent AE terms not present in source data.
- NEVER use non-CDISC controlled terminology where controlled terminology
  is defined.
- NEVER deduplicate AE records without source verification.
- Abort if raw AE data or the study configuration is missing.
```

Sources and standalone references

1. The Required column reflects this study's mapping specification, not SDTMIG Core designations (Required/Expected/Permissible).

2. Clinical Data Interchange Standards Consortium. Study Data Tabulation Model Implementation Guide: Human Clinical Trials, v3.4. https://www.cdisc.org/standards/foundational/sdtmig (accessed 2026-07-26).

3. The complete 59-file rule corpus is included in the versioned reproducibility release (doi:10.5281/zenodo.21614975).
