# Supplementary material for: Workflow Topology–Guided Skill Loading for Agentic AI in Clinical Trial Data Pipelines: Sequential Exploratory Evaluation
Source: JMIR Form Res. 2026 Oct 20;10:e100769. doi: 10.2196/100769 (PMC13732176; doi:10.2196/100769)
Supplement: Supplementary file 9 [file formative_v10i1e100769_app5.pdf]

Adaptive Priority Scheduler Algorithm

Standalone supplementary material. Author and institution cover-page identifiers have been removed; the scientific content below is retained from the authoritative source PDF.

```
graph-declared global skills.
   2  Classify: Assign each candidate to its highest-priority directed-adjacency band: CURRENT, SUCCESSOR,
      PREDECESSOR, or GLOBAL.

   3  Rank: Use band weight multiplied by a capped hub-degree bonus; break ties alphabetically. Query relevance is
      not used.
   4  Allocate: Service CURRENT candidates toward an initial 40% target, then process all unprocessed candidates
      against one shared remaining budget.

   5  Truncate: If a candidate does not fit, strip frontmatter, strip code blocks, then apply a character cutoff; drop
      targets below 100 estimated tokens.
   6  Emit: Inject the ordered loaded or truncated skill content and record per-skill provenance.

   Formal Specification

    INPUT: graph G, active node v*, token budget B, reserved tokens R
    1 S <- skills(v*) U skills(successors(v*)) U skills(predecessors(v*))
         U graph_global_skills
    2 classify each s in S by first match: CURRENT, SUCCESSOR,
         PREDECESSOR, GLOBAL
    3 priority(s) <- band_weight(s) *
         [1 + min(0.3, 0.03 * hub_degree(s))]
    4 rank S by descending priority, then alphabetical skill name
    5 A <- max(0, B - R); used <- 0
    6 current_target <- min(CURRENT demand, floor(0.4 * A))
    7 allocate ranked CURRENT candidates until serviced >= current_target;
         mark those candidates processed
    8 allocate every unprocessed candidate in ranked order against A - used
    9 return loaded and truncated candidates with audit metadata
   Proximity Band Definitions
   Band   Name   Weight                   Executed definition
     0   CURRENT  1.0  Skills bound directly to the active node; serviced first and exempt from the single-skill cap.
     1  SUCCESSOR 0.6  Skills bound to immediate downstream successors of the active node.
     2 PREDECESSOR 0.4 Skills bound to immediate upstream predecessors of the active node.
                       Graph-declared global skills, plus unmatched candidates as a defensive fallback; admitted only
if
     3    GLOBAL  0.2
                       budget remains.
```

Token Budget Allocation

After reserving any reference or distilled-principle tokens, CURRENT candidates are serviced first toward a target equal to the smaller of their aggregate demand and 40% of available tokens. The implementation then processes all unprocessed candidates - including any remaining CURRENT candidates - against one shared remaining budget. It does not create proportional budgets for bands 1-3.

```
current_target = min(current_demand, floor(0.4 * available_budget))
priority(s) = band_weight(s) * [1 + min(0.3, 0.03 * hub_degree(s))]
```

The compatibility argument named query_match is ignored in the scheduler source recorded by the component-comparison checkpoint. No semantic or lexical query bonus contributed to ranking.

Allocate Procedure

Let L be the shared remaining budget. If L is zero, the candidate is dropped. Otherwise its target is the smaller of its estimated tokens and L. For a non-CURRENT skill, the target is additionally capped at 25% of the available budget. A full-fitting candidate is loaded. A non-fitting candidate with a target of at least 100 estimated tokens is truncated by stripping YAML frontmatter, stripping code blocks, and applying a character cutoff at four characters per estimated token; smaller targets are dropped. CURRENT candidates are exempt from the per-skill cap but can still be truncated or dropped if the overall budget is exhausted. No CURRENT truncation or drop occurred in the 30 corrected-APS runs.

Execution Status Across Experiments

In the original experiment, a skill-name normalization issue classified every candidate as GLOBAL. The band weights, CURRENT service target, and hub ranking were therefore not applied, and the framework arm performed node-bound rule injection with uniform alphabetical priority.
The experiment runner restored normalization and constructed the current/successor/predecessor/global candidate union. Across its 30 corrected-APS runs, stored audits show CURRENT content in 30/30 runs, SUCCESSOR content in 27/30, PREDECESSOR content in 3/30, and GLOBAL candidates present but dropped under the budget in 30/30. All four bands occurred among candidates, but only three contributed loaded content, and no single context loaded all four bands.

Implementation Reference

Executed file: scripts/context-loader.py
Corrective-checkpoint scheduler SHA-256:
ed0125d6db267d7456df58b53d0687b9e
ad076d5ffae484380916c582d88154f
Source code: https://github.com/yanmingyu92/csp-workflow-engine

Sources and standalone references

1. Implementation source: scripts/context-loader.py and the versioned corrective-experiment runner identified in the manuscript.
2. The pseudocode documents an architecture property and does not assert regulatory compliance.
