# Supplementary material for: Workflow Topology–Guided Skill Loading for Agentic AI in Clinical Trial Data Pipelines: Sequential Exploratory Evaluation
Source: JMIR Form Res. 2026 Oct 20;10:e100769. doi: 10.2196/100769 (PMC13732176; doi:10.2196/100769)
Supplement: Supplementary file 10 [file formative_v10i1e100769_app6.pdf]

Computational Environment, API Costs, and Token Variation

Standalone supplementary material. Author and institution cover-page identifiers have been removed; the scientific content below is retained from the authoritative source PDF.

1. Computational Environment

All experiments were conducted using the following computational environment:

- Agent system: Claude Code command-line interface agent harness (Anthropic; invoked as claude -p)routingtoaGLM-5 backbone model(ZhipuAI)viaanAnthropic-compatible API endpoint. The backbone model identity is recorded in the released experiment metadata (agent models: ["glm-5"]).
- Evaluation LLM (judge): DeepSeek (deepseek-chat API endpoint), used for LLM-as-a-judge Tier 1 (pairwise) and Tier 2 (multidimensional) scoring, and for the 2 persona-conditioned evaluation panels. The judge belongs to a different model family from the generation backbone.
- Compute: Cloud-based API calls; no local GPU required.
- Operating system: Windows 11 Pro.
- Python version: 3.12.
- Statistical analysis: NumPy and SciPy 1.12+ (bootstrap, exact sign-flip tests).

2. API Cost Details

Token costs include both prompt and completion tokens.

- Primary experiment (90 agent runs + judge scoring): US $16.04, as recorded in the experiment metadata (agent cost plus judge cost).
- Full evaluation program(includingthe2panelevaluationruns, pairwisecomparisons, and pilot runs): approximately US $49.
- Mean cost per execution: US $0.163 (unconstrained baseline) vs US $0.182 (graph-constrained framework), a difference of +11.6%, attributable to the injected skill context.

3. Per-Task Token Cost Variation

Per-execution cost varies by task because response length and the volume of loaded skill context differ across pipeline layers. Tasks at layers 0 and 2 (protocol parsing, SDTM mapping) load larger controlled-terminology skill files and produce longer structured outputs, placing them at the upper end of the per-execution cost range; validation tasks (layer 3) sit at the lower end. The complete per-run token counts (input and output) and per-run costs for all 90 runs are included in the released experiment JSON in the public repository, enabling exact reproduction of the cost figures reported in Table 1 of the manuscript.

Sources and standalone references

1. Environment and cost records are stored in the experiment metadata and result checkpoints cited in the manuscript.

2. Python Software Foundation. Python 3.12 documentation. https://docs.python.org/3.12/ (accessed 2026-07-26).
