# Supplementary material for: Workflow Topology–Guided Skill Loading for Agentic AI in Clinical Trial Data Pipelines: Sequential Exploratory Evaluation
Source: JMIR Form Res. 2026 Oct 20;10:e100769. doi: 10.2196/100769 (PMC13732176; doi:10.2196/100769)
Supplement: Supplementary file 11 [file formative_v10i1e100769_app7.pdf]

LLM-as-a-Judge Prompt Templates, Persona Specifications, and Randomization Procedures

Standalone supplementary material. Author and institution cover-page identifiers have been removed; the scientific content below is retained from the authoritative source PDF.

1. Scope

This appendix reproduces, verbatim, the judge prompt templates executed in the experiments, together with the persona specifications used by the 2 compliance evaluation panels and the presentation-orderrandomizationprocedures. Alltemplatesarealsoincludedinthereleasedsource code, and every judged call in the component comparison records the SHA-256 hash of its rendered prompt in the result file.

2. Tier 2: Multidimensional Scalar Judge Prompt (executed template)

Placeholders: {task description}=thetaskprompt;{domain criteria}=node-specificmandatory/recommended criteria drawn from the regulatory pattern library (empty string if none); {response} = the agent output under evaluation. In the original experiment the response placeholder received the first 4000 characters; in the component comparison 's complete-response rejudgment it received the full stored response (see Multimedia Appendix 12).

```
You are a senior regulatory submission quality reviewer with 15+ years of CDISC experience.
Evaluate this clinical data programming output on THREE separate dimensions.

TASK: {task_description}
{domain_criteria}

OUTPUT TO EVALUATE:
{response}
Score EACH dimension independently from 1-5:

**COMPLETENESS**: Are all requested items addressed?
 1 = Missing most items
 2 = Some items addressed, significant gaps
 3 = All requested items present at conceptual level
 4 = All items + implementation details (derivation logic, code snippets)
 5 = All items + edge cases + validation checks
**TERMINOLOGY**: Are CDISC/regulatory terms used correctly?
 1 = No regulatory terminology
 2 = Some terms but incorrect usage
```

```
3 = Correct variable names and concepts (USUBJID, SDTM, ADaM)
 4 = + Codelist references (C66731), controlled terminology, ISO 8601
 5 = + Cross-domain traceability, IG version references

**STRUCTURE**: Is the output machine-readable and actionable?
 1 = Unstructured text only
 2 = Basic formatting (headers, bullets)
 3 = Tables with clear organization
 4 = YAML/JSON/code with derivation logic
 5 = Production-ready spec with schema validation
Respond with ONLY valid JSON, no markdown:
{"completeness": <1-5>, "terminology": <1-5>, "structure": <1-5>, "reasoning": "<brief explanation
   -→ >"}
```

The overall score is the unweighted mean of the 3 dimensions. Judge calls used temperature 0.

3. Tier 1: Pairwise Judge Prompt (executed template)

```
You are a senior regulatory submission quality reviewer with 15+ years of CDISC experience.
Compare these two outputs for the SAME clinical data programming task.
TASK: {task_description}

{domain_criteria}

--- OUTPUT A ---
{response_a}
--- OUTPUT B ---
{response_b}

Which output better addresses the regulatory task? Consider:
- Completeness (all items addressed?)
- Regulatory terminology accuracy (CDISC CT, variable names)
- Actionability (can a programmer implement from this?)
- Structure (tables, YAML, code blocks)
Respond with ONLY valid JSON, no markdown:
{"winner": "A" or "B" or "tie", "reason": "<brief explanation>", "confidence": "high" or "medium"
   -→ or "low"}
```

4. Randomization Procedures

Original experiment (single orientation). For each pairwise comparison, the assignment of the two conditions to positions A and B was randomized by a uniform coin flip (flip = random() < 0.5); the winner label was mapped back to the underlying condition after judging. Condition labelswerenevershowntothejudge. Becauseeachpairwaspresentedinonlyoneorientation,these win rates are descriptive; the component comparison s bidirectional diagnostic later demonstrated strong presentation-order sensitivity (58/90 logical pairs order-discordant), and single-orientation pairwise results are therefore not used as confirmatory evidence.

(bidirectional). Everyoneofthe90replicate-matchedcomparisons was presented in both AB and BA orientations (180 presentations). A logical pair was counted as

a decisive win only when both orientations normalized to the same winning condition; otherwise it was labeled order-discordant. Full presentation-level records, including rendered-prompt and response hashes, are in the released result file.

5. Panel Persona Specifications

Eachofthe2complianceevaluationpanelscomprised3persona-conditionedevaluatorperspectives. The persona preambles prepended to the evaluation instructions were:

- Clinical Data Manager (CDM):“Youareaseniorclinicaldatamanagerwithexpertisein EDC systems, CDASH/SDTM mapping, data cleaning, CRF design, MedDRA/WHODrug coding, and CDISC controlled terminology governance.”
- Statistical Programmer (SP): “You are a senior statistical programmer with expertise in SAS/R/Python programming for ADaM/TFL production, CDISC ADaM IG v1.3, macro development, Pinnacle 21 validation, and QC/double-programming.”
- Regulatory Specialist (RS): “You are a regulatory submission specialist with expertise in ICH E3/E6(R2)/E8/E9 guidelines, FDA 21 CFR Part 11, eCTD Module 5 structure, Define.xml 2.0/2.1 specification, and GxP compliance.”

PanelevaluatorsreceiveddeidentifiedoutputpairsinrandomizedA/Border,scoredeachoutput on the 3 dimensions (1–5), issued a forced pairwise choice (A, B, or tie), and provided free-text feedback. Per-pair panel scores and agreement statistics are given in Multimedia Appendix 11.

6. Provenance

The executed templates in Sections 2 and 3 are drawn verbatim from the released experiment runner. The component comparison's scoring protocol additionally records, for every call: the requested and returned judge model identifiers, the API system fingerprint, rendered-prompt SHA-256, response SHA-256, token counts, and cost. See Multimedia Appendix 12 for the component comparison's provenance summary.

Sources and standalone references

1. Executed templates and prompt hashes are recorded by the experiment runners and result artifacts described in the manuscript.

2. DeepSeek-AI. DeepSeek-V3 Technical Report. arXiv:2412.19437. doi:10.48550/arXiv.2412.19437.
