# Supplementary material for: Workflow Topology–Guided Skill Loading for Agentic AI in Clinical Trial Data Pipelines: Sequential Exploratory Evaluation
Source: JMIR Form Res. 2026 Oct 20;10:e100769. doi: 10.2196/100769 (PMC13732176; doi:10.2196/100769)
Supplement: Supplementary file 12 [file formative_v10i1e100769_app8.pdf]

Full-Response Reanalysis of the Original Bundled Experiment

Locked secondary reanalysis of 90 stored responses (10 tasks x 3 conditions x 3 replicates). The task is the inferential unit; 3 replicates are averaged within each task-condition cell before paired inference.

Integrity repair and estimand

The original scalar and pairwise judge paths received only the first 4000 characters; 57/90 stored responses exceeded that limit. All complete stored responses were scored with the pinned deepseek-v4-flash nonthinking JSON protocol at temperature 0. The estimand is the mean of 10 paired task differences for the bundled framework minus the bare agent. Confidence intervals use 10,000 paired task-bootstrap resamples, and P values use the exact 2-sided sign-flip distribution over the 10 task differences.

Outcome	Bare mean	Framework mean	Difference	95% CI	Exact P	Paired dz
Overall	3.5897	4.4667	+0.8770	[0.6333, 1.1320]	0.001953	2.07
Structure	3.6333	4.6667	+1.0333	[0.7000, 1.4000]	0.001953	1.73
Terminology	3.4333	4.2667	+0.8333	[0.6000, 1.0667]	0.001953	1.97
Completeness	3.7000	4.4667	+0.7667	[0.5667, 1.0000]	0.001953	1.98

All 10 overall task differences were positive. Leave-one-task-out overall estimates ranged from +0.80 to +0.94. Holm adjustment across the 3 component dimensions yielded P=.005859 for structure, terminology, and completeness. Framework plus principles minus framework was -0.0547 (95% CI -0.2327 to 0.1453; exact P=.65625), providing no detectable incremental benefit.

Protocol sensitivity and bidirectional pairwise order results

Under the obsolete first-4000-character prefix protocol, the framework-minus-bare-agent overall difference was +0.47 (95% CI 0.14 to 0.82; exact P=0.0234; paired dz=0.81), preserving the direction of the full-response result with a smaller magnitude. In the bidirectional pairwise sensitivity analysis, each of the 30 replicate-matched framework-versus-bare-agent pairs was judged in both AB and BA order: 21 pairs favored the framework in both orders, 0 favored the bare agent in both orders, and 9 were order-discordant. Pairwise judgments were descriptive only and were not used for inferential claims.

Interpretation boundary

This comparison bundled the domain prompt, curated rule access, and node-bound injection. A revision-stage source audit found that the intended banded scheduler was not exercised. The result is therefore secondary evidence for the bundled intervention and cannot identify graph routing as the causal mechanism. Prefix-versus-full differences are protocol sensitivity, not a causal truncation effect, because response coverage and judge-version/time effects are inseparable.

Sources and standalone references

1. Machine-readable source: paper/jmir_ai/analysis_original_full/analysis-summary.json; SHA-256 9b19003d004509eabb12fdee9b7320e15482ffd6d2603065c3b73207427359a4.

2. Complete-response checkpoint: experiments/results/experiment_original_rejudged_full_outputs.json; SHA-256 4587417f6fe07b11de6f3aa39a3c63cf3fe85ea3f3d59d78dc3a0cd338cd93e1. Recomputing the full analysis from this checkpoint reproduces the reported statistics exactly.

3. Locked protocol SHA-256: 869e41e424383a0ab6ea8c6f07ae493d743d85ff1514e69c4fd863124a66ef4b.
