# Supplementary material for: Workflow Topology–Guided Skill Loading for Agentic AI in Clinical Trial Data Pipelines: Sequential Exploratory Evaluation
Source: JMIR Form Res. 2026 Oct 20;10:e100769. doi: 10.2196/100769 (PMC13732176; doi:10.2196/100769)
Supplement: Supplementary file 13 [file formative_v10i1e100769_app9.pdf]

Multimedia Appendix 9

Blinded Expert–LLM-Judge Concordance Study

Jaime Yan
Harrisburg University of Science and Technology

1. Purpose and prespecification

This study addresses the reviewer requirement that the LLM-based compliance scores be checked against practicing domain experts. A practicing statistical programmer (SP), blinded to condition, independently rescored complete stored outputs of the six-condition component comparison using the identical 3-dimension rubric executed by the automated judge (Multimedia Appendix 7) . The design, blinding procedure, sampling rule, and every statistic below were frozen in a written protocol *before* any scoring occurred; the sealed identity key (SHA-256 `3ab22775f3642e1f156ee262bbad640c17f81ba943540e1bf4813ee38a8eaa5e`) was opened only after the completed scoring sheet was returned. One disclosed protocol adjustment : the protocol anticipated reporting in a later round or follow-up study; the decision to report in this revision was made after results were known. No result value was altered by that decision.

2. Rater, materials, and blinding

The rater is a senior statistical programmer (10 years of late-phase oncology statistical programming; SDTMIG v3.4, ADaMIG v1.3, Define-XML v2.1, and Pinnacle 21 expertise) with no authorship or financial interest in this study. Materials were a census of all 60 task-condition cells of the component comparison (10 tasks $\times$ 6 conditions; one stored replicate per cell selected by a seeded deterministic draw; seed 42), presented in a random order (seed 20260726) under blind IDs with all condition, context, and model metadata stripped. Each blinded file contained exactly what the LLM judge saw: the task statement, the task's domain-specific mandatory/recommended criteria, and the complete stored response. The rater scored completeness, terminology, and structure (1–5 anchored scales identical to the judge rubric) and provided a free-text comment for every item (60/60). The rater reported a total scoring time of approximately 60 minutes. A post-scoring confirmation documented that the reported time was accurate and that no AI assistance was used.

3. Primary results: concordance with the locked judge scores (n=60)

The overall mean difference is compatible with no systematic bias. Terminology—the dimension closest to objective CDISC fact-checking—shows substantial agreement. Structure shows insufficient agreement, and the expert scored structure systematically lower than the judge (CI excludes 0), consistent with a production-deployability standard that the judge's format-presence anchors do

Dimension	Weighted κ	Spearman ρ	Expert−judge Δ [95% CI]	Band
Terminology	0.61	0.61	$+0.07$ $[-0.10, +0.23]$	substantial
Completeness	0.54	0.50	-0.10 $[-0.23, +0.03]$	moderate
Structure	0.35	0.31	-0.23 $[-0.40, -0.07]$	insufficient
Overall (3-dimension mean)	ICC(2,1)=0.60	0.50	-0.09 $[-0.21, +0.03]$	moderate

Table 1: Expert-versus-judge agreement on the identical 60 outputs. Quadratic-weighted Cohen κ; ICC(2,1) is two-way random effects, absolute agreement, single rater; bootstrap CIs use 10,000 item-level resamples. Prespecified interpretation bands: $\kappa \geq 0.61$ substantial, 0.41–0.60 moderate, ≤ 0.40 insufficient. All values are reported unconditionally per protocol.

not capture. This divergence independently supports the routing-sensitive follow-up's design decision to replace judge-scored structure with machine-verifiable schema validators. Both raters used only scores 3–5 on this panel; range restriction attenuates κ, so rank agreement may be modestly understated.

4. Secondary results: does the expert reproduce the condition pattern?

Expert task-paired contrast		Mean diff	Exact P	Holm P	LLM counterpart
APS	− domain prompt only	+0.37	.074	.22	+0.43 (Holm P=.012)
	− flat loading	+0.27	.16	.31	+0.05 (Holm P=.78)
	− random loading	+0.13	.63	.63	+0.19 (Holm P=.42)

Table 2: Expert task-level paired contrasts (10 tasks; exact 2-sided sign-flip tests; Holm adjustment across the 3 contrasts). The expert data use 1 replicate per cell and a single rater (the LLM analysis used 3 replicates), so lower power is expected. All 3 expert point estimates are directionally positive and, as in the LLM analysis, the APS-versus-domain-prompt contrast is the largest; the relative ordering of the flat and random controls is reversed compared with the judge. Nothing in the expert data contradicts the routing-null conclusion.

5. Figures

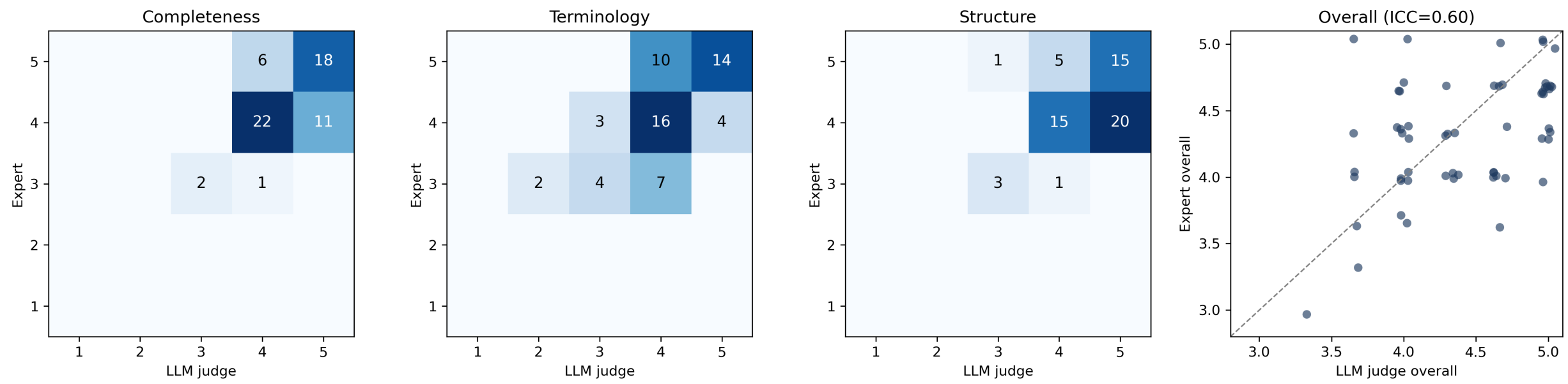

Figure A9-1. Item-level agreement: per-dimension confusion matrices (expert vs judge) and overall scatter with identity line.

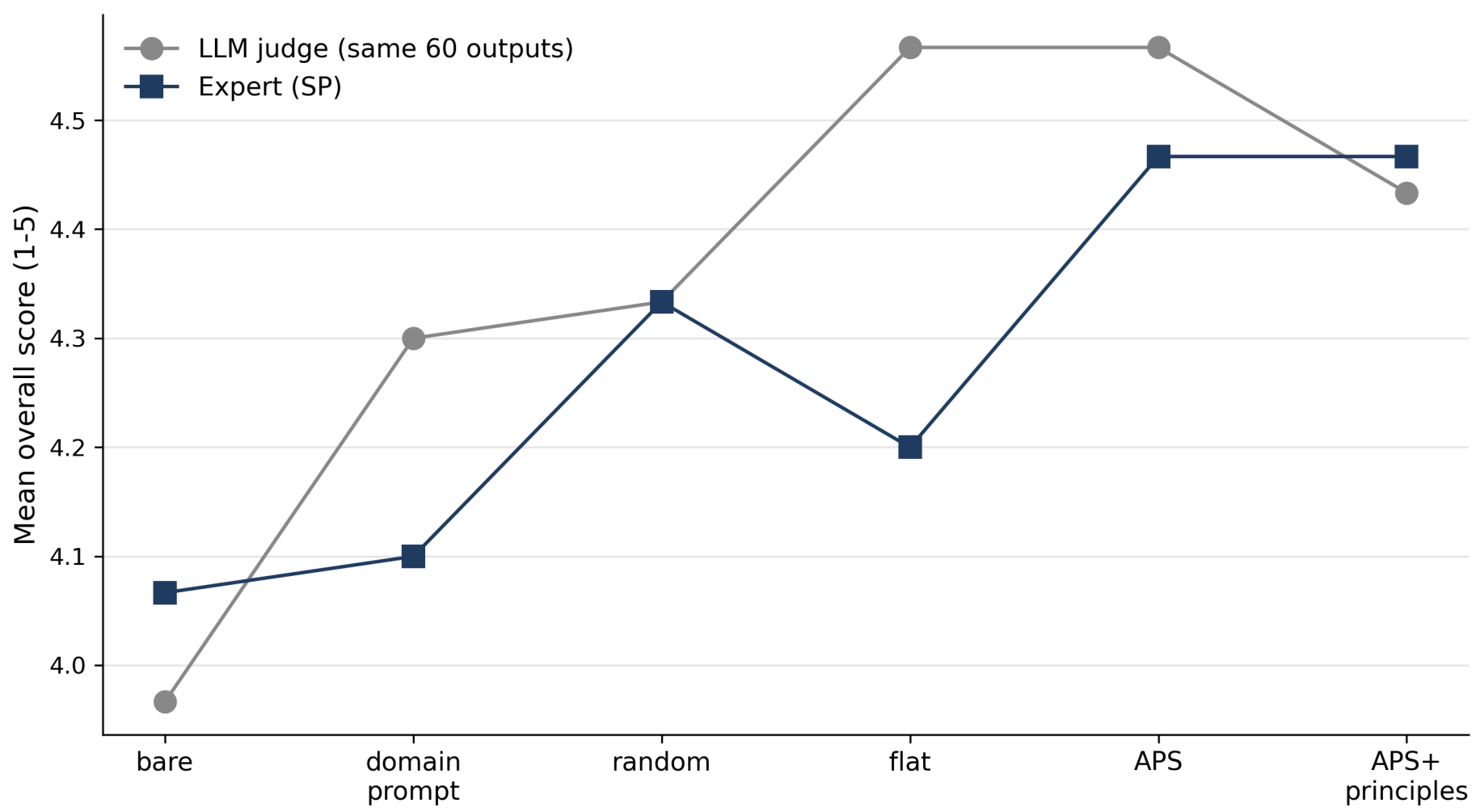

Figure A9-2. Mean overall score by condition on the identical 60 outputs: both raters place the bare agent lowest and show no routed-versus-nonrouted-corpus advantage.

6. Limitations

(1) Single rater; inter-rater reliability is not estimable, and additional independent professionals would be needed to establish expert consensus. (2) Score range restriction (3–5) attenuates agreement coefficients. (3) Stored responses can self-reveal their condition through content; this exposure is identical for the expert and the LLM judge. (4) These results validate rank-ordering agreement on one synthetic panel and one judge version; they do not establish expert-level validity of any individual score.

7. Provenance

Frozen protocol, blinded package, sealed key (hash above), scoring sheet, frozen analyzer, machine-readable results, and both figures are included in the versioned reproducibility release referenced in the Data Availability statement.
