# Supplementary material for: Workflow Topology–Guided Skill Loading for Agentic AI in Clinical Trial Data Pipelines: Sequential Exploratory Evaluation
Source: JMIR Form Res. 2026 Oct 20;10:e100769. doi: 10.2196/100769 (PMC13732176; doi:10.2196/100769)
Supplement: Supplementary file 14 [file formative_v10i1e100769_app14.docx]

Multimedia Appendix 14

# Topology-Dependent Stress Panel and Boundary-Condition Diagnostics

This appendix provides the detailed tables, listing, and figure underlying the constructed topology-dependent stress-panel result. It is intended to make the main manuscript's conditional statement auditable without forcing implementation-level detail into the main Results. The panel is a mechanism stress test under fixed author-constructed conditions; it is not an external replication, a randomized factorial estimate of a graph main effect, or evidence of regulatory readiness.

# A14.1 How the Main Claim Should Be Read

**Table A14-1. Claim-to-evidence boundary.**

| **Statement** | **Status in this study** | **Reason** |
| --- | --- | --- |
| Topology can recover dependency-linked evidence absent from query vocabulary | Supported as a mechanism demonstration | Graph-only required-evidence recall was 1.000 versus 0.500 for full-corpus BM25 on the constructed panel |
| Topology improves outcomes when lexical retrieval already reaches the evidence | Not supported | On the lexically reachable panel, BM25 recall was 0.958 and graph+BM25 minus BM25 was +0.009 (95% CI -0.028 to 0.046; Holm *P*=.67) |
| The stress-panel contrast isolates a causal graph effect | Not supported | Graph-only and BM25-only differed in score term, selector/admission policy, and context volume |
| The stress result establishes downstream substantive-quality superiority | Not established | Provenance contributed 40%-50% of the score; the target-collapsed provenance-excluded sensitivity was not positive at *P*<.05 |
| The result generalizes to clinical operations, other graphs, or other backbones | Not tested | One author-built graph/corpus, one generator backbone, synthetic tasks, and no operational outcomes |

# A14.2 Constructed Panel and Selection Policies

The panel contained 18 task IDs, 6 arms, and 3 generation repetitions (324 stored scalar-score rows). Repetitions were averaged within each task-arm cell before task-level comparisons. Each prompt named an active workflow node, while the required source was at least 1 hop away. The prompt omitted the required skill name and tokens from the required section heading. Seventeen of 18 required sections were first-document-order, low-priority sections. The task/gold map was constructed from the same graph and corpus, and an offline oracle verified separation before generation.

**Table A14-2. Executed selection policies and evidence-context volume.**

| **Arm** | **Graph signal** | **Lexical signal** | **Candidate/admission policy** | **Mean canonical tokens (range)** |
| --- | --- | --- | --- | --- |
| Graph only | Yes | No | Reachable skills ranked by hop band; deterministic skill-round-robin/document order | 2157.28 (1994-2380) |
| Typed graph only | Yes, with typed-edge priority | No | Graph-only policy with typed-edge priority | 2135.11 (1926-2319) |
| Graph+BM25 | Yes | Yes | Graph-expanded candidates followed by BM25 chunk ranking | 2481.89 (1992-2947) |
| Full-corpus BM25 | No | Yes | Lexical ranking over the full corpus | 4000.00 (4000-4000) |
| Random | No | No | Seeded random selection at a fixed 4000-token target | 4000.00 (4000-4000) |
| Flat | No | No | Flat loading at an 8000-token ceiling | 8000.00 (8000-8000) |

These cells were not a shared-pipeline factorial design. Context length and admission logic changed with the graph/BM25 signals; therefore, diagonal graph-only versus BM25-only is descriptive and cannot be interpreted as toggling only graph use.

# A14.3 Complete Arm Results

**Figure A14-1.** Complete constructed stress-panel arm results. Panel A shows mean deterministic task score after averaging 3 repetitions within task-arm cells. Panel B shows required-evidence recall across 54 task-repetition cells per arm. The same 18 author-constructed tasks were used for all arms. Exact values are in Table A14-3. The figure is descriptive because selectors and evidence-context volumes differed across arms.

**Table A14-3. Per-arm score and required-evidence recall.**

| **Selection policy** | **Task mean score (0-1)** | **Required-evidence recall** |
| --- | --- | --- |
| Graph only | 0.715 | 1.000 |
| Graph only with typed-edge priority | 0.663 | 1.000 |
| Full-corpus BM25 | 0.367 | 0.500 |
| Graph expansion + BM25 | 0.352 | 0.444 |
| Flat loading at 8000 tokens | 0.089 | 0.111 |
| Random loading at a fixed 4000-token target | 0.096 | 0.019 |

**Table A14-4. Five code-declared paired contrasts.**

| **Paired contrast** | **Mean difference** | **Paired-bootstrap 95% CI** | **Exact sign-flip *P*** | **Paired *d*_z_** | **Post hoc Holm *P*** |
| --- | --- | --- | --- | --- | --- |
| Graph only - full-corpus BM25 | +0.3481 | 0.1778 to 0.5296 | .001953 | 0.893 | .005859 |
| Graph only - flat | +0.6259 | 0.4630 to 0.7778 | .0000305 | 1.793 | .000122 |
| Graph only - random | +0.6185 | 0.4963 to 0.7407 | .00000763 | 2.264 | .0000381 |
| Typed graph only - graph only | -0.0519 | -0.1000 to 0.0000 | .125 | -0.470 | .250 |
| Graph+BM25 - full-corpus BM25 | -0.0148 | -0.1556 to 0.0778 | 1.000 | -0.056 | 1.000 |

The five-contrast family was not prospectively locked. Holm values are therefore reported only as a post hoc robustness check. They do not convert the author-constructed panel into a confirmatory study.

# A14.4 Analysis-Unit and Outcome Sensitivity

Four task IDs reused adam-adsl-builder > Key Variables, and 2 reused adam-traceability-checker > Philosophy. In addition, the total score combined one substantive criterion with a provenance-citation criterion worth 40% on 16 tasks and 50% on 2 tasks. Raw criterion records and response text were not retained, so provenance exclusion could only be reconstructed as bounds.

**Table A14-5. Sensitivity of graph-only minus full-corpus BM25.**

| **Analysis and endpoint** | **Unit count** | **Mean difference** | **Exact sign-flip *P*** | **Interval or bound** |
| --- | --- | --- | --- | --- |
| Total score, task IDs | 18 | +0.3481 | .001953 | 95% *t* interval 0.1543 to 0.5420 |
| Total score, unique required-evidence targets | 14 | +0.2512 | .015625 | 95% *t* interval 0.0702 to 0.4321 |
| Provenance-excluded content score, bounded reconstruction by task ID | 18 | +0.2407 to +0.2778 | .046875 to .03125 | Exact value unavailable |
| Provenance-excluded content score, bounded reconstruction by unique target | 14 | +0.1310 to +0.1429 | .25 | Exact value unavailable |

The result is robust as an evidence-access manipulation check: the graph-only arm included all required sections and BM25 included half. The stronger claim that topology improved downstream substantive output after accounting for repeated targets and provenance weighting is not established.

# A14.5 Task and Required-Evidence Listing

**Table A14-6. Constructed task inventory.**

| **Task ID** | **Active node** | **Required skill** | **Required heading** | **Hop distance** |
| --- | --- | --- | --- | --- |
| v9h01-adae-needs-adsl-keyvars | adam-adae | adam-adsl-builder | Key Variables | 1 |
| v9h02-adae-needs-ae-philosophy | adam-adae | sdtm-ae-mapper | Philosophy | 1 |
| v9h03-adtte-needs-adsl-keyvars | adam-adtte | adam-adsl-builder | Key Variables | 1 |
| v9h04-adlb-needs-lb-philosophy | adam-adlb | sdtm-lb-mapper | Philosophy | 1 |
| v9h05-tables-needs-adsl-keyvars | tfl-table-generation | adam-adsl-builder | Key Variables | 1 |
| v9h06-tables-needs-shell-philosophy | tfl-table-generation | tfl-shell-reviewer | Philosophy | 1 |
| v9h07-figures-needs-adsl-keyvars | tfl-figure-generation | adam-adsl-builder | Key Variables | 1 |
| v9h08-define-needs-p21-philosophy | define-xml-sdtm | p21-validator | Philosophy | 1 |
| v9h09-define-needs-draft-structure | define-xml-sdtm | define-draft-builder | Define.xml Structure | 1 |
| v9h10-define-val-needs-builder-philosophy | define-xml-validation | define-xml-builder | Philosophy | 1 |
| v9h11-p21-needs-dm-philosophy | p21-sdtm-validation | sdtm-dm-mapper | Philosophy | 1 |
| v9h12-p21adam-needs-traceability-philosophy | p21-adam-validation | adam-traceability-checker | Philosophy | 1 |
| v9h13-ae-needs-spec-philosophy | sdtm-ae-mapping | spec-builder | Philosophy | 1 |
| v9h14-dm-needs-rawval-philosophy | sdtm-dm-mapping | data-validator | Philosophy | 1 |
| v9h15-adsl-needs-sap-philosophy | adam-adsl | sap-parser | Philosophy | 1 |
| v9h16-esub-needs-sdrg-philosophy | esub-package-assembly | sdrg-writer | Philosophy | 1 |
| v9h17-adrg-needs-traceability-philosophy | adam-reviewer-guide | adam-traceability-checker | Philosophy | 1 |
| v9h18-tflqc-needs-table-philosophy | tfl-qc-validation | tfl-table-generator | Philosophy | 1 |

The 18 identifiers represent 14 unique (required skill, required heading) targets. Rephrased prompts, repeated gold packages, criteria, and model repetitions are repeated measurements rather than independent evidence families.

# A14.6 Reproducibility and Artifact-Retention Boundary

**Listing A14-1. SHA-256 values recorded at the statistical audit.**

experiments/results/v9_graph_only_live_evaluated.json

8d24c120a616a16d88fcbfafa85ca7363dacc0e5c4a55741ebeccacd750292b0

experiments/results/v9_graph_only_live_analysis.json

a6195e678293497402473c603906c42867cf08e3c351a599fbad1ea5e167a953

experiments/analyze_v9_graph_only_live.py

12e756138e9c409266d8549d205e36e1e420f1699b75368e03120820bf87c7fd

experiments/run_experiment_v9_graph_only.py

50c6a09a9cf84a06301f6304a2f7fe6a972ee67885c0acf26001ee218851f98c

experiments/v9_graph_only_retrieval.py

15a9cbde24a2552905a8ae20fb515583b1a28b99e55b84d6acc0780002e0f226

experiments/config/experiment_v9_routing_hostile_tasks.yaml

aaaabd626fa502f8d13c2a63745eb25c21514f1aeae88b55fdbd9ef214a4a8bb

These hashes identify the inspected working-copy state. At audit time the files were ignored or untracked under the repository's experiments/ ignore rule; they are not represented as an immutable public release.

The evaluated result bundle retained 324 scalar scores, response hashes, and enough identifiers to reproduce the reported task-arm aggregates. It did not retain all raw response text, transmitted contexts, criterion-level records, returned model identifiers, timestamps, attempts/retries, or full manifests. Consequently, the original outputs cannot be independently rescored and the provenance-excluded endpoint cannot be reconstructed exactly.

# A14.7 Design Implications

A future confirmatory evaluation should use independently authored dependency/evidence families; freeze tasks and gold evidence before graph mapping; use a shared candidate universe, chunker, serializer, selector, token budget, and tie-breaker in a true 2×2 graph-by-lexical design; randomize masked complete blocks; retain all contexts, responses, criterion records, model identifiers, timing, retries, and manifests; separate substantive quality from provenance; and require replication across a second backbone and independently constructed workflow graph. These are future-validation requirements, not prerequisites for reporting the present bounded exploratory mechanism result.
