# Supplementary material for: Workflow Topology–Guided Skill Loading for Agentic AI in Clinical Trial Data Pipelines: Sequential Exploratory Evaluation
Source: JMIR Form Res. 2026 Oct 20;10:e100769. doi: 10.2196/100769 (PMC13732176; doi:10.2196/100769)
Supplement: Supplementary file 15 [file formative_v10i1e100769_app15.docx]

Multimedia Appendix 15: Retained Records of Generative AI Use in Manuscript Preparation

# A15.1 Purpose and Scope

JMIR asks authors who use generative artificial intelligence (AI) tools in manuscript preparation to retain the original prompts and responses and to submit them as a multimedia appendix. This appendix inventories the records that were retained, reproduces representative records verbatim, and states plainly what was not retained. No record is reconstructed, summarized into a synthetic dialogue, or simulated: where complete transcripts do not exist, this appendix says so.

As stated in the Use of AI Statement of the main text, this appendix covers only the generative AI tools used to prepare the manuscript. The AI system evaluated in the study is described in the Methods of the main text; its computational environment and model identifiers are recorded in Multimedia Appendix 6 and in the archived result metadata of the reproducibility release, and it plays no further role in this appendix.

# A15.2 Tools, Roles, and Version Information

**Table A15-1.** Preparation tools, their roles, and version-information retention.

| **Tool** | **Developer** | **Role in manuscript preparation** | **Version retained?** |
| --- | --- | --- | --- |
| Grammarly (generative AI mode) | Grammarly, Inc. | Language polishing | No; browser-based subscription service, version not recorded |
| OpenAI Codex | OpenAI | Proposing language edits; cross-document consistency checks | No; version identifier not recorded |
| Claude Code | Anthropic | Rebuilding document files; verification scripting | No; command-line interface version identifier not recorded |

The three preparation tools were subscription services that update continuously, and their version identifiers were not recorded at the time of use. They are reported as unavailable rather than reconstructed. The retained records below therefore identify sessions by date, session identifier, and working context rather than by product version.

# A15.3 Inventory of Retained Records

Two classes of preparation records were retained in the project repository.

First, Claude Code sessions were configured with a local logging hook that writes an automated work log at session end, recording the session identifier, timestamp, working directory, git branch, and a machine-generated summary of files added or modified. These logs evidence when agent-assisted file rebuilding and verification work occurred and what it touched; they do not contain per-turn prompts or model responses.

**Table A15-2.** Retained automated Claude Code session work logs.

| **Record** | **Session ID** | **Timestamp** | **Working directory (relative)** | **Branch** | **Changes** |
| --- | --- | --- | --- | --- | --- |
| session-20260415-8ae27526.md | 8ae27526-9d8e-4e25-b02f-30a73654cef9 | 2026-04-15 13:07 | paper/ | main | Logged in work log |
| session-20260415-7e5c45b7.md | 7e5c45b7-ea32-42f5-adbe-29c6e42c478b | 2026-04-15 13:58 | paper/ | main | Logged in work log |
| session-20260415-c5d7cfeb.md | c5d7cfeb-56f7-44db-83e2-f64108128dc4 | 2026-04-15 16:46 | paper/ | main | Logged in work log |
| session-20260716-92634276.md | 92634276-1175-4397-a754-89b26facb551 | 2026-07-16 11:21 | repository root | main | Logged in work log |
| session-20260716-d8f2ad30.md | d8f2ad30-a967-442c-b023-1c8d73a3864d | 2026-07-16 11:21 | repository root | main | Logged in work log |
| session-20260716-2045024a.md | 2045024a-7915-4a3d-9423-6ee9c4a33cd9 | 2026-07-16 11:21 | repository root | main | Logged in work log |
| session-20260716-25b40021.md | 25b40021-747d-44a6-a27d-4a17a8b97daf | 2026-07-16 11:22 | repository root | main | Logged in work log |
| session-20260726-341ff17b.md | 341ff17b-b2e6-4f56-bbb1-9dbdc8b624d8 | 2026-07-25 21:11 | paper/jmir_ai | codex/jmir-corrected-experiment-v5 | Logged in work log |

Second, the verbatim task documents given to OpenAI Codex and Claude Code, together with dated working notes and completion reports from those sessions, were retained in the repository plan directory. These include the instruction texts supplied to the tools (execution prompts, task plans, and frozen protocols) and session outputs such as working notes and reports, dated by filename.

**Table A15-3.** Retained task, prompt, and working records from the preparation-tool sessions.

| **Record (verbatim filename)** | **Date** | **Content descriptor** |
| --- | --- | --- |
| jmir_corrected_experiment_design_20260716.md | 2026-07-16 | Component-comparison redesign instructions |
| jmir_corrected_experiment_implementation_20260716.md | 2026-07-16 | Component-comparison implementation prompt |
| jmir_revision_audit_20260716.md | 2026-07-16 | Revision self-audit instructions |
| jmir_revision_round2_fixes_20260722.md | 2026-07-22 | Package-level fix plan |
| jmir_aps_gxp_optimization_experiment_20260724.md | 2026-07-24 | Context-efficiency experiment instructions |
| jmir_aps_gxp_v6_execution_prompt_20260724.md | 2026-07-24 | Execution prompt for the efficiency experiment |
| jmir_aps_gxp_v7_routing_sensitive_experiment_20260725.md | 2026-07-25 | Lexically reachable retrieval experiment instructions |
| jmir_v7_bm25_gap_evaluation_20260725.md | 2026-07-25 | Evidence-gap evaluation instructions |
| jmir_v7_oracle_retrieval_error_decomposition_20260725.md | 2026-07-25 | Offline evidence-audit instructions |
| jmir_v8_typed_graph_retrieval_optimization_20260725.md | 2026-07-25 | Typed-edge variant instructions |
| jmir_v8_typed_graph_retrieval_optimization_research_notes_20260725.md | 2026-07-25 | Typed-edge variant research notes |
| jmir_systematic_polish_strategy_20260725.md | 2026-07-25 | Systematic language-polish strategy and workflow |
| jmir_v8_evaluation_20260726.md | 2026-07-26 | Stress-panel evaluation instructions |
| jmir_ai_submission_remediation_20260726_task_plan.md | 2026-07-26 | Submission-package remediation task plan |
| jmir_ai_submission_remediation_20260726_notes.md | 2026-07-26 | Remediation working notes |
| jmir_ai_submission_remediation_20260726_final_report.md | 2026-07-26 | Remediation completion report |
| expert_validation_protocol_20260726.md | 2026-07-26 | Frozen protocol for the blinded expert concordance study (Multimedia Appendix 9) |
| jmir_v9b_preregistered_protocol_20260727.md | 2026-07-27 | Frozen analysis protocol |
| v9b/ (directory) | 2026-07-27 | Supporting frozen configuration files |
| .Codex/evals/v7-oracle-retrieval-error-decomposition.md | 2026-07 | Evaluation-design record |
| .Codex/evals/v8-typed-graph-retrieval-optimization.md | 2026-07 | Evaluation-design record |

# A15.4 Representative Retained Record, Reproduced Verbatim

Table A15-4 reproduces the most recent retained session work log (2026-07-25, during manuscript revision) verbatim. Within field values, line breaks are rendered as semicolons, local username path components are shortened, decorative pictographs in the source headings are omitted, and the log's trailing auto-generated CLAUDE.md change-details subtable is omitted; no other alteration is made. The remaining seven logs follow the same format.

**Table A15-4.** Verbatim automated session work log, session 341ff17b (2026-07-25).

| **Field** | **Verbatim content** |
| --- | --- |
| Title | Work Log - jmir_ai |
| Session ID | 341ff17b-b2e6-4f56-bbb1-9dbdc8b624d8 |
| Time | 2026/07/25 21:11:56 |
| Directory | .../csp-workflow-engine/paper/jmir_ai |
| Session changes, branch | codex/jmir-corrected-experiment-v5 |
| Session changes, files | M csp-skills/layer-2-sdtm/sdtm-ae-mapper/SKILL.md; M csp-skills/layer-2-sdtm/sdtm-dm-mapper/SKILL.md; M csp-skills/layer-3-sdtm-qc/define-draft-builder/SKILL.md; M csp-skills/layer-3-sdtm-qc/p21-validator/SKILL.md; M csp-skills/layer-4-adam/adam-adsl-builder/SKILL.md; M csp-skills/layer-5-tfl/tfl-demographics/SKILL.md; M csp-skills/layer-5-tfl/tfl-qc-validator/SKILL.md; M csp-skills/layer-5-tfl/tfl-table-generator/SKILL.md; M csp-skills/layer-6-submission/define-xml-builder/SKILL.md; M csp-skills/layer-6-submission/define-xml-validator/SKILL.md; .Codex/ (added); agents/ (added); generators/ (added); hooks/ (added); manuscripts_clean/ (added); mcps/ (added); methodology/ (added); plan/jmir_aps_gxp_v6_execution_prompt_20260724.md (added); plan/jmir_aps_gxp_v7_routing_sensitive_experiment_20260725.md (added); plan/jmir_corrected_experiment_design_20260716.md (added); ... (39 more changes omitted) |
| Change summary | Added 49; Modified 10; Deleted 0 |
| Next steps (auto-generated) | Uncommitted changes detected (59 files); CLAUDE.md memory needs updating (129 source files changed); run /update-memory to sync latest memory |

# A15.5 Retention Boundaries

The following boundaries are disclosed so that the completeness of this record can be evaluated directly:

- Complete per-turn dialogue transcripts (every prompt and every model response of every interactive session) were not exported from the tools' session stores at the time of use and are therefore not available for any of the three preparation tools. No transcript is reconstructed or simulated in this appendix.
- No Grammarly records of any kind exist: it is a browser-based subscription service, and no export was retained.
- Product version identifiers of the three preparation tools were not recorded (Table A15-1).
- What is retained: the eight automated session work logs (Table A15-2), the verbatim task and prompt documents listed in Table A15-3, and the successive manuscript versions, including the tracked-changes comparison against the originally submitted manuscript that accompanies this revision.
- The retained records are available from the author on reasonable request.
